# The Human Orphan Nuclear Receptor Tailless (TLX, NR2E1) Is Druggable

**DOI:** 10.1371/journal.pone.0099440

**Published:** 2014-06-17

**Authors:** Cindy Benod, Rosa Villagomez, Carly S. Filgueira, Peter K. Hwang, Paul G. Leonard, Guillaume Poncet-Montange, Senapathy Rajagopalan, Robert J. Fletterick, Jan-Åke Gustafsson, Paul Webb

**Affiliations:** 1 Department of Genomic Medicine, Houston Methodist Research Institute (HMRI), Houston, Texas, United States of America; 2 Department of Biophysics and Biochemistry, University of California San Francisco, San Francisco, California, United States of America; 3 Department of Biochemistry and Molecular Biology, MD Anderson Cancer Center, University of Texas, Houston, Texas, United States of America; 4 Center for Biomolecular Structure and Function, MD Anderson Cancer Center, University of Texas, Houston, Texas, United States of America; 5 University of Houston Center for Nuclear Receptors and Cell Signaling, Houston, Texas, United States of America; University of Geneva, Switzerland

## Abstract

Nuclear receptors (NRs) are an important group of ligand-dependent transcriptional factors. Presently, no natural or synthetic ligand has been identified for a large group of orphan NRs. Small molecules to target these orphan NRs will provide unique resources for uncovering regulatory systems that impact human health and to modulate these pathways with drugs. The orphan NR tailless (TLX, NR2E1), a transcriptional repressor, is a major player in neurogenesis and Neural Stem Cell (NSC) derived brain tumors. No chemical probes that modulate TLX activity are available, and it is not clear whether TLX is druggable. To assess TLX ligand binding capacity, we created homology models of the TLX ligand binding domain (LBD). Results suggest that TLX belongs to an emerging class of NRs that lack LBD helices α1 and α2 and that it has potential to form a large open ligand binding pocket (LBP). Using a medium throughput screening strategy, we investigated direct binding of 20,000 compounds to purified human TLX protein and verified interactions with a secondary (orthogonal) assay. We then assessed effects of verified binders on TLX activity using luciferase assays. As a result, we report identification of three compounds (ccrp1, ccrp2 and ccrp3) that bind to recombinant TLX protein with affinities in the high nanomolar to low micromolar range and enhance TLX transcriptional repressive activity. We conclude that TLX is druggable and propose that our lead compounds could serve as scaffolds to derive more potent ligands. While our ligands potentiate TLX repressive activity, the question of whether it is possible to develop ligands to de-repress TLX activity remains open.

## Introduction

Human nuclear receptors (NRs) are ligand-dependent transcriptional factors that control a myriad of biological and disease processes [1]. Many NRs are regulated by lipophilic molecules that can be theoretically exchanged with a drug of choice [2]. Upon ligand binding, recruitment of a range of positive (coactivators) or negative (corepressors) regulatory proteins are key steps in ligand-induced activation or repression, respectively, of the transcription of NR target genes. NR ligands have been used in a number of important therapeutic areas, including breast and prostate cancers, skin disorders, and diabetes [2]. However, a large number of NRs have no identified natural or synthetic ligand and are referred to as “orphan” NRs [3]. Development of drugs to target this orphan NR subclass could open up new approaches for a variety of human diseases.

Tailless (TLX, NR2E1) is an orphan NR expressed exclusively in the brain [4]. TLX is located in the nucleus, suggesting that it is engaged in transcriptional regulation under basal conditions and it has been shown to act as a transcriptional repressor when bound to the *p21*, *pten*, *gfap* or *S100b* promoters [5], [6]. While no crystal structure of TLX is yet available, sequence homology analysis suggests that it possesses a canonical DNA binding domain, a hinge region and a Ligand Binding Domain (LBD). Uncharacteristically, TLX lacks an N-terminal domain containing the ligand-independent activation function (AF-1).

TLX is expressed in neural stem cells (NSC) and rapidly dividing neural progenitor cells and is linked to maintaining NSC in an undifferentiated proliferative state in developing and adult brains [6]–[17]. Shi *et al.* [8] demonstrated that TLX-expressing cells can proliferate, self-renew, and differentiate into all three major neural cell types (neurons, astrocytes, oligodendrocytes) *in vitro*. Conversely, *Tlx*-null cells isolated from adult *Tlx*-null brains failed to proliferate and reintroduction of TLX into *Tlx*-null cells rescued their ability to proliferate and self-renew [8]. In adult brain, TLX represses the cyclin-dependent kinase inhibitor *p21* and the tumor suppressor *pten* expression via interactions with the histone deacetylase 5 (HDAC5) and the lysine specific demethylase 1 (LSD1) [5], [6], [15]. Accordingly, knockdowns of HDAC and LSD1 or inhibition of HDAC activity led to marked induction of *p21* and *pten* gene expression, thereby reducing NSC proliferation and allowing NSC differentiation [5], [6], [15]. TLX also maintains undifferentiated NSC state by transrepressing differentiation-related genes, such as *gfap* and *S100b* [15]. Given the essential roles of TLX in NSC maintenance and self-renewal, small molecules that reverse TLX repressive activities could trigger neurogenesis and combat neurodegenerative diseases [17].

TLX is also expressed in Brain Tumor Stem Cells (BTSC), leading to the hypothesis that brain tumors arise from aberrant NSC [18]. In this context, Liu *et al.* [19] demonstrated that NSC-specific overexpression of *Tlx* induces NSC expansion and glioma-like lesions in adult mouse brain which progress to invasive glioma when p53 function is lost. TLX is also overexpressed in various human glioma cell lines and glioma stem cell lines [20]. Ectopic expression of TLX in U87MG glioma cells accelerates cell proliferation and transformation with concomitant elevation of cyclin D1 [20]. Further, a series of gene profiling studies [21]–[26] showed that TLX is overexpressed in various types of human glioma, including neuroblastoma, astrocytoma, and ependymona, and its expression was correlated with a poor prognosis. These results suggest that TLX is a valuable diagnostic marker to detect NSC-derived brain tumors and a potential therapeutic target to inhibit their development [20].

While the biological significance of TLX is clear, lack of TLX ligands has impaired our ability to validate TLX as a druggable therapeutic target. Identification of lead compounds that interact with TLX will be a critical step for determining whether it may be possible to develop small molecule modulators of TLX activity. Development of synthetic ligands for other NRs [2], and orphan NRs [27], raises hopes that it may be possible to discover ligands useful for controlling TLX activity [17]. Presently, however, TLX LBD organization is not clear and it is not known whether TLX possesses druggable ligand binding sites.

Here, we report results, which suggest that TLX is indeed druggable. Development of homology models of the TLX LBD suggests that TLX belongs to an emerging class of orphan NR with non-canonical LBDs and also points toward the existence of a large open Ligand Binding Pocket (LBP). Medium throughput screening approaches combining direct binding and cell-based assays allow us to identify the first described ligands targeting human TLX LBD and prove that TLX is druggable. Moreover, these ligands enhance TLX repressive actions in cultured cells confirming that it is possible to modulate TLX activity with small molecules. Our ligands could serve as scaffolds to derive more potent and specific TLX ligands to pursue further characterization of this receptor.

## Materials and Methods

### Homology Modeling of Human TLX LBD

The amino-acid sequence of the published TLX LBD (aa 187–385) was retrieved from the TrEMBL/Swiss-Prot databank (http://expasy.org/Sprot). This sequence was searched against the PDB database (http://www.pdb.org/pdb/home/home.do) using two metaservers (TOME V2, http://bioserv.cbs.cnrs.fr/and the Sali laboratory server, http://www.salilab.org). At the time of that study, in both cases, the known X-ray structures of two human NRs: COUP-TFII (COUP Transcription Factor II, pdb 3CJW [28]) and RXRα (Retinoid X Receptor α, pdb 1RDT [29]) were selected since they possess the highest consensus scores and share reasonable homologous sequence identity of 38% and 35%, respectively. Multiple structural sequence alignment was obtained using ClustalW2 (http://www.ebi.ac.uk/Tools/msa/clustalw2/) and included 10 supplementary sequences of NR LBDs with crystal structures available (data not shown). Manual refinements of this alignment were performed using V.I.T.O software [30]. For creation of the homology model of TLX LBD in an agonist conformation and to obtain an image of a potential LBP for TLX, a chimera template including both the crystal structures of COUP-TFII and RXRα was used. Homology models of TLX LBD in an agonist conformation using the alignment and the chimera template were generated with MODELLER 9v8 (freely available, http://www.salilab.org/modeller/, [31]). The three resulting models were evaluated using the Eval123D server (http://bioserv.cbs.cnrs.fr/HTML_BIO/valid.html) and further assessed using the WinCoot program. The top ranked model was selected. As shown in the Ramachandran plot (Fig. S1), 94.52% (186 residues) of the residues are in preferred regions, 4.57% (9 residues) are in the allowed regions, 2 residues are outliers (1.02%).

### Recombinant Protein Expression and Purification

cDNA fragments encoding human TLX LBD (a.a. 187–385) were cloned into pET-46 Ek/LIC vector (Novagen) containing the N-terminal His_6_-tag followed by a TEV protease cleavage site according to manufacturer’s protocol and checked by sequencing (ACGT, Inc.). Recombinant protein was expressed in BL21 (DE3) star cells (Life Technologies) using standard methods (induction with 0.1 mM IPTG followed by 24 hours culture at +16°C), and purified using Ni^2+^-NTA affinity column (Qiagen) followed by size exclusion chromatography (HiLoad 16/60 Superdex 200, GE Healthcare, Life Sciences) in buffer containing 20 mM Tris pH 8.0, 150 mM NaCl, 5 mM DTT, 10% glycerol (v/v) and 2 mM CHAPS.

Human estrogen receptor (ER) β His_6_-ERβ LBD (residues 261–530) was expressed in BL21 (DE3) cells (Life Technologies) and human LXRβ LBD (Liver X Receptor beta, a.a. 226–460) was expressed from a pET-46 Ek/LIC vector (Novagen) containing the N-terminal His_6_-tag followed by TEV protease cleavage site in BL21 (DE3) star cells (Life Technologies). Induction strategy and purification methods were similar to TLX LBD.

### Mass Spectrometry

Identity and purity of recombinant NR LBDs were evaluated using mass spectrometry (HMRI proteomics core). Gel filtration fractions containing purified proteins were diluted 2-fold with 50 mM ammonium bicarbonate (pH 8.0), followed by reduction and alkylation of cysteine residues as follows. DTT was added to a final concentration of 5 mM and the solution heated to 60°C for 30 min; iodoacetamide was added to 15 mM and the solution incubated at room temperature in the dark for an additional 30 min. Sequencing grade trypsin (Promega) was added at a final ratio of 1∶50 (wt∶wt) enzyme:substrate and the solution incubated overnight at 37°C. The following morning another bolus of trypsin was added at 1∶50 and incubated for an additional 4 hours. Resulting peptides were diluted in water/0.1% formic acid for injection onto the LC/MS system. Briefly, tryptic peptides liberated from the digestion were resolved by liquid chromatography reverse phase gradient separation (eluted with increasing acetonitrile solvent concentration) on a 75 µm×250 mm BEH C-18 UPLC column (1.7 µm particle size; waters Corp.) using a NanoAcquity UPLC system (Waters Corp.) before being introduced via a nanoelectrospray ionization source using positive ion mode into a Synapt mass spectrometer (Waters Corp.) for analysis. Tandem MS analysis was accomplished in the Synapt instrument utilizing a parallel ion fragmentation strategy using alternating low and high collision energies in successive MS scans, termed MS^E^. LC/MS instrument control and data acquisition was accomplished using MassLynx software (Waters Corp., v4.1). Proteins were identified from their tryptic peptide molecular ions as well as their corresponding product ion spectra produced upon high energy CID fragmentation by a computerized protein database search strategy of resulting MS^E^ data [32], [33] against the Uniprot human proteome database (version 2012_11; 20,227 entries) after lockmass correction using ProteinLynx Global Server (PLGS v2.3, Waters Corp.) software. The search algorithm was limited to a 1% false discovery rate by use of both reverse and randomized decoy target databases in the search strategy. Protein quantification was carried out as described [33], [34] using the Identity^E^ algorithm of the PLGS software.

### Analytical Ultracentrifugation

Sedimentation equilibrium experiments were performed at 4°C using a Beckman XL-I instrument with AnTi60 rotor using Epon double sector centerpieces with sapphire windows. All samples were prepared in 20 mM Tris-HCl, 150 mM NaCl, 5 mM DTT, 10% (v/v) glycerol, 1% (v/v) DMSO and 2 mM CHAPS at pH 8.0. Sedimentation equilibrium profiles were recorded every 7 hours for a total of 70 hours at each of three rotor speeds: 11,000 r.p.m., 18,000 r.p.m. and 22,000 r.p.m. Data analysis was performed using Sedphat 10.58d [35], [36]. The protein partial specific volume and solvent density were calculated using Sednterp 1.09. The reported error values were determined using an F-statistics error mapping approach for 95% confidence intervals.

### Differential Scanning Fluorimetry (DSF)/Thermofluor

Protein stability was assessed with DSF in absence or presence of test compounds in a LightCycler 480 II qRT-PCR Detection System (Roche) in 384-well format. Sypro-Orange dye (Life Technologies) was used to monitor fluorescence, with filters for fluorescence excitation at 465 nm and for fluorescence emission at 610 nm. The DSF spectra for purified TLX LBD (2.5 µM in 20 µL final volume) was recorded using TBS (Tris Buffer Saline pH 8.0) as a screening buffer with added Sypro-Orange dye (1/2000 dilution, Life Technologies), in the presence of individual compounds (500 µM) or 5% DMSO (control). Tested sample mixtures were heated from 25°C to 96°C at a rate of 0.05°C/sec with 11 acquisitions per °C. Each compound was tested in duplicates. The melting temperature (T_m_) for each sample was deduced by the ThermoQ Analytical software (HMRI) from the first derivative of the corresponding denaturation curve generated by the LightCycler 480 software. The same protocol was applied to investigate the stability of purified ERβ and LXRβ LBDs in presence or absence of the compounds. The optimal final concentrations for these experiments were 0.02 mg/mL for ERβ LBD and 0.20 mg/mL for LXRβ LBD. All DSF-based analyses were performed using freshly purified protein.

### Chemical Libraries

The libraries used in this study were the NIH clinical Collections I and II (www.nihcclinicalcollection.com), FDA-approved Drug Library from Selleck chemicals (www.selleckchem.com), Maybridge HitFinder library (www.maybridge.com), Prestwick Chemical Library (www.prestwickchemical.com), and National Cancer Institute (NCI) mechanistic, diversity and natural products sets.

### Biolayer Interferometry

For these experiments, human TLX LBD cDNA (a.a. 187–385) was recloned into pET-Duet (Novagen) vector containing an N-terminal His_6_-tag followed by the AVITAG sequence. Cloning was accomplished with a standard double digest cloning protocol after PCR according to manufacturer’s protocol (Avidity), and the plasmid was sequenced (ACGT, Inc). Recombinant protein was co-expressed in BL21(DE3) star cells with Biotin Ligase (BirA) plasmid (gift from Dr. Kristopher Kuchenbecker, Fletterick Laboratory, UCSF) using standard methods in LB media supplemented with 50 µM Biotin (Fisher Scientific, induction with 0.1 mM IPTG followed by 24 hours cell culturing at +16°C). Recombinant protein was purified using Ni^2+^-NTA affinity column (Qiagen) followed by size exclusion chromatography (HiLoad 16/60 Superdex 200, GE Healthcare, Life Sciences) in buffer containing 20 mM Tris pH 8.0, 150 mM NaCl, 5 mM DTT, 10% glycerol (v/v) and 2 mM CHAPS. Measurements with the Octet Red 384 instrument (*FortéBio Inc.*, Menlo Park, CA) were performed with TBS supplemented with 2 mM CHAPS as a screening buffer at RT. Super-streptavidin (SSA) biosensors (FortéBio) were coated in a solution containing 2 µM of TLX LBD at 25°C to a loading signal of 10 nm. A duplicate set of sensors which serve as reference surfaces were incubated in 200 µM solution of blocked biotinylated Streptavidin protein (Scripps Laboratories) to a loading signal of about 5.5 nm as described by manufacturer’s protocol (FortéBio). Binding of the individual compounds (100 µM) or 1% DMSO (solvent control) was measured over immobilized TLX LBD target biosensors and reference surfaces. Corrected binding response sensorgrams were recorded and analyzed. Data analysis on the *FortéBio Octet RED* instrument was performed using the *FortéBio* data analysis software. The analysis accounts for non-specific binding, background, and signal drift and minimizes well based and sensor variability.

Compounds able to bind TLX LBD were further evaluated in dose-response experiments with concentrations ranging from 100 µM to 400 nM in triplicates in three independent experiments. The corresponding equilibrium dissociation constants (K_D_) were determined using steady state analysis of compounds binding affinities, assuming 1∶1 ligand - protein stoichiometry.

### Transactivation Assays

We cloned TLX LBD (residues 187–385) downstream of a Gal4 DNA-binding domain (Gal4-DBD) in the vector pBIND according to manufacturer’s protocol (Promega). The luciferase reporter pGL4.35 [*luc2P*/9X*GAL4*UAS/Hygro] vector was obtained from Promega. pBIND-TLX LBD was fully sequenced (ACGT, Inc.).

HeLa cells were maintained in D-MEM High Glucose (Hyclone) supplemented with 10% charcoal:dextran stripped FBS (Hyclone) and 1x Pen/Strep antibiotics (Life Technologies). Transient transfections were carried out in batches on 100,000 cells seeded into 12-well tissue culture plates. The co-transfections were performed using the TransFectin Lipid reagent (Bio-Rad) with approximately 10 ng/well of Gal4 DBD (gift from Phuong Nguyen, Fletterick Laboratory, UCSF) or GAL4-TLX LBD, 10 ng/well of renilla luciferase gene (Promega) for internal control and 200 ng/well of pGL4.35 reporter gene (containing an upstream Gal4 Upstream Activator Sequence and the luciferase gene, Promega). Approximately, 3 h after transfections, cells were treated with compounds of interest at different concentrations, or solvent (DMSO, 0.1%) in DMEM containing no fetal bovine serum or antibiotics. After a 16-h incubation, luciferase activities were assessed using Dual Luciferase assay reagent (Promega). Luciferase activities were calculated by normalizing firefly luciferase to renilla luciferase signal. Normalized luciferase activities were then represented relative to control (DMSO-treated cells). Cells transfected with Gal4 DBD vector served as a control for TLX independent effects.

For the transactivation assays involving human PNR (Photoreceptor cell-specific Nuclear Receptor, NR2E3) LBD, we used an already described pBIND-PNR LBD (residues 192–410, [37]) plasmid (gift from Dr. H. Eric Xu, Van Andel Institute). The transient co-transfections of HeLa cells with vectors encoding either Gal4DBD or pBIND-PNR LBD fusion (both at 50 ng/well), pGL4.35 reporter gene (200 ng/well) and renilla luciferase gene (50 ng/well, internal control) were performed in batches of 10^5^ cells seeded into 12-well tissue culture plates. Twenty-four hours after the transfections, cells were treated with either DMSO (0.1%, control) or compounds ccrp1, ccrp2 or ccrp3 at 10 µM. Following 24 h incubation, luciferase activities in each well were assessed as described above. Cells transfected with Gal4 DBD vector served as a control for PNR independent effects.

We cloned human RXRα LBD (residues 225–462) in the vector pBIND as described above. Transient co-transfections of HeLa cells with vectors encoding either Gal4DBD or Gal4 DBD-RXRα LBD fusion (both at 10 ng/well), pGL4.35 reporter gene (200 ng/well) and renilla luciferase gene (10 ng/well, internal control) were performed in batches of 10^5^ cells seeded into 12-well tissue culture plates. The transfections were done using TransFectin Lipid Reagent (Biorad). Three hours after the transfections, cells were treated with either DMSO (0.1%, control) or compounds ccrp1, ccrp2 or ccrp3 at 10 µM, in the absence or the presence of 9-cis retinoic acid (RA, 100 nM). Following 24 h incubation, luciferase activities in each well were assessed as described above. Cells transfected with Gal4 DBD vector served as a control for RXR independent effects.

For the transactivation assays with the GAL4 DBD-COUP-TFII LBD plasmid ([28], gift from Dr. H. Eric Xu, Van Andel Institute), we transiently transfected in batches of 10^5^ HeLa cells seeded into 12-well tissue culture plates vectors encoding either Gal4 DBD or Gal4 DBD-COUP-TFII LBD fusion (both at 100 ng/well), pGL4.35 reporter gene (200 ng/well) and renilla luciferase gene (100 ng/well, internal control). At 24 h after the transfections, cells were treated with either DMSO (0.1%, control) or compounds ccrp1, ccrp2 or ccrp3 at 10 µM, in the absence or the presence of 9-cis retinoic acid (RA, 5 µM). Following 24 h incubation, luciferase activities in each well were assessed as described above. Cells transfected with Gal4 DBD vector served as a control for COUP-TFII independent effects.

For ERβ, transient cotransfections of HeLa cells with vectors encoding either Gal4 DBD or Gal4 DBD-ERβ LBD fusion (gift from Dr. Stefan Andersson, University of Houston, deceased), both at 10 ng/well, constructs for Gal4-E1B promoter linked to a luciferase reporter gene (200 ng/well) and renilla luciferase gene (10 ng/well, internal control) were performed in batches of 10^5^ cells seeded into 12-well tissue culture plates. At 24 h after the transfections, cells were treated with either DMSO (0.1%, control) or ccrp1; ccrp2 and ccrp3 at 10 µM, in the presence or absence of estradiol (E2, 100 nM), in the medium containing no fetal bovine serum. Following 24 h incubation, luciferase activities in each well were assessed as described above. Cells transfected with Gal4 DBD vector served as a control for ERβ independent effects.

All measurements were performed in triplicates and repeated in three independent experiments. Prism 6 was used to calculate EC_50_.

## Results

### TLX Ligand Binding Pocket (LBP) should Accommodate Ligands

**Figure 1:**
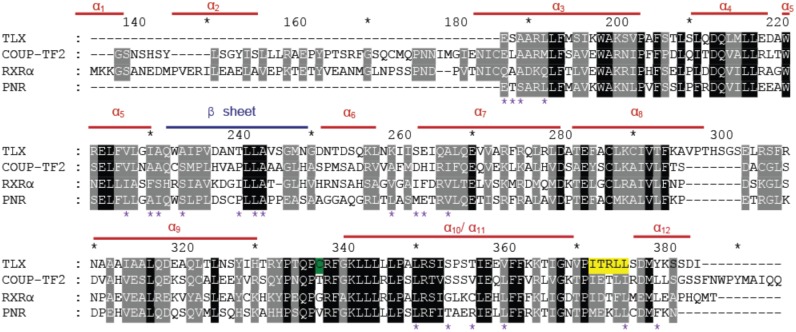
Sequence alignment of TLX LBD with COUP-TFII, RXRα and PNR LBDs. The nomenclature of the helices and β-sheet is indicated. Predicted residues belonging to the LBP are indicated by pink stars. Residues involved in the binding of TLX corepressors are highlighted in yellow. An exposed cysteine C338 is highlighted in green.

**Figure 2:**
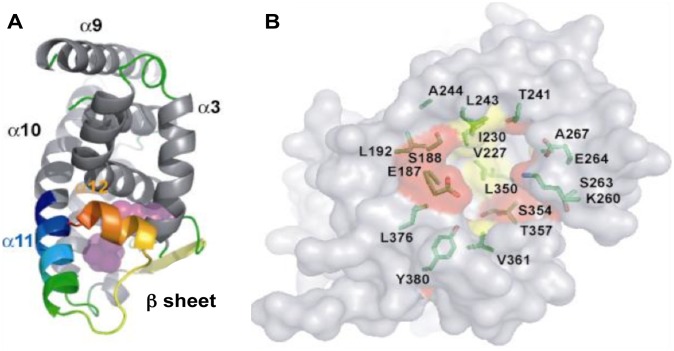
Models of TLX LBD and TLX LBP. A. This panel represents TLX LBD in a putative agonist conformation. Helix α11 is in blue/green, helix α12 in orange/yellow. The LBP appears in pink circles behind helix α12. B. This panel illustrates the key amino-acids inside the LBP, hydrophobic residues are in yellow, hydrophilic residues in red.

Until this study, no natural or synthetic ligands have been identified for TLX and no crystal structures of the TLX LBD are yet available. To determine whether TLX might possess a druggable LBP, we created a homology model of TLX LBD in a transcriptionally active conformation mainly using the closely related RXRα LBD as a template (Materials and Methods). The primary sequence of TLX LBD comprises amino-acids 187–385. Similar to DAX1 (dosage-sensitive sex reversal, adrenal hypoplasia critical region, on chromosome X, gene 1; NR0B1) [38], SHP (Small Heterodimer partner, NR0B2) [39] and PNR [37], the structural alignment used for designing the TLX LBD model (Fig. 1) revealed that it is shorter than other NR LBDs and lacks the first helices α1 and α2. The alignment also reveals a unique insertion (aa. 296–310) between the helices α8 and α9. Despite these notable differences, the predicted TLX LBD model (Fig. 2 panel A) shares most aspects of the canonical NR LBD helical sandwich fold [1]. In this model, helix α11 is positioned in continuity with α10 and helix α12 folds over the LBD delimiting a putative LBP.

Crystal structures of NR LBDs with agonists reveal that ligand is buried in a predominantly hydrophobic pocket composed of residues from helices α3, α5, α11, α12, the β-sheet and loops L6–7 and L11–12. The LBP is situated in the bottom part of the LBD in a flexible region. Superposition of the RXRα structure upon the TLX LBD model predicts that TLX harbors a LBP that is large enough (around 500 Å^3^) to accommodate ligands (Fig. 2 panel B). The pocket should not be filled with amino-acid side chains as previously observed with the nuclear receptor Nurr1 (Nuclear Receptor Related 1 protein, NR4A2) [40]. The predicted TLX LBP nevertheless differs significantly from other NR LBPs in that the absence of helices α1 and α2 increases the size and solvent exposure. Further, the predicted TLX LBP can be divided into 2 parts. There is a buried portion that comprises a strong hydrophobic environment exemplified by residues V227, I230, A267, L350 and similar to LBPs of other NRs. Additionally, there is a solvent exposed portion created by the absence of helices α1 and α2 that is comprised of mostly polar residues such as E187, K260, E264, S354, T357, Y380. The multiple alignment analysis (including 10 sequences of NR LBDs) revealed that these polar residues are not conserved amongst the NR superfamily and are specific to TLX, and the other member of its subfamily PNR, which shares the highest sequence homology with TLX. Based on this observation, we speculate that TLX could accommodate ligands with hydrophobic features to enter the LBP and polar moieties that interact with solvent and polar residues near the opening of the pocket.

### Biochemical Characterization of the Recombinant Purified TLX LBD

**Figure 3:**
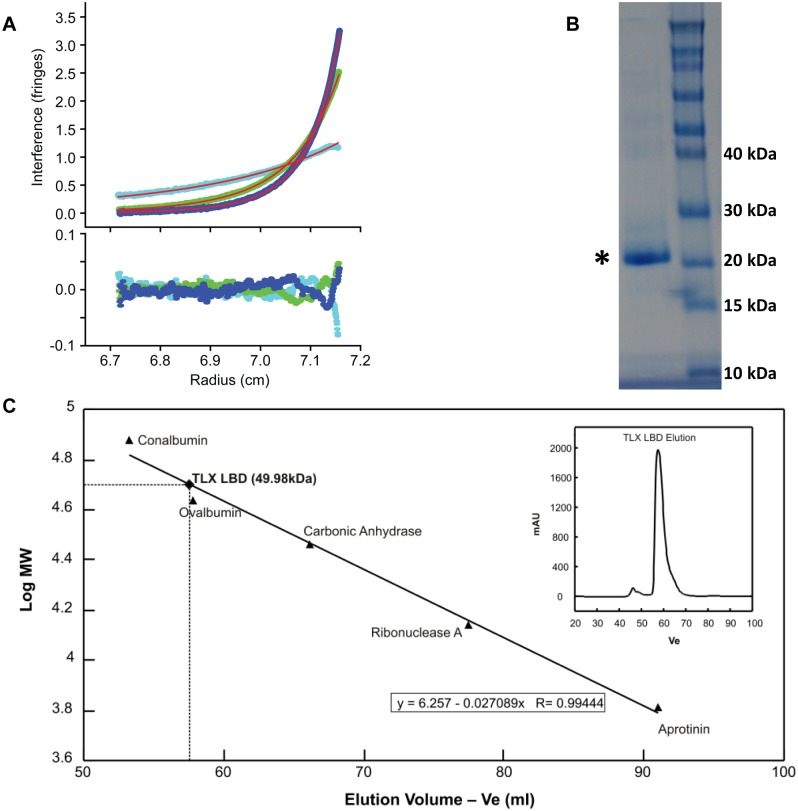
Biochemical characterization of purified TLX LBD. A. Analytical ultracentrifugation sedimentation analysis on purified TLX LBD. Analytical ultracentrifugation sedimentation equilibrium profiles were recorded at 4°C after 70 hour incubation at three rotor speeds: 11,000 r.p.m. (cyan), 18,000 r.p.m. (green) and 22,000 r.p.m. (blue), for a 10 µM TLX sample, dissolved in 20 mM Tris-HCl, 150 mM NaCl, 5 mM DTT, 10% (v/v) glycerol, 1% (v/v) DMSO and 2 mM CHAPS at pH 8.0. The upper panel shows the sedimentation equilibrium profiles with the lines of best fit shown in red. The best fit was obtained for a monomer-dimer equilibrium model with a Kd of 10 µM. 95% confidence interval limits for this dissociation constant were determined to be 4 µM<Kd<24 µM. The lower panel provides, for each dataset, the residuals for the data fitting. This is an illustration on how accurate the fitting actually is. B. This panel represents the SDS page gel for the pooled fractions of purified TLX LBD protein after size-exclusion chromatography (first column, shown with the asterisk). The protein ladder appears in the second column. In presence of this denaturing gel, TLX LBD runs at a molecular weight of 25 KDa, corresponding to the molecular weight for the monomeric TLX LBD. C. This panel is an illustration of the size-exclusion calibration curve. TLX LBD elutes at a volume corresponding to a dimer. In the inset window is shown the size-exclusion chromatogram for TLX LBD. TLX LBD elutes as a sharp symmetric peak.

Recombinant TLX protein was expressed and purified as described in Materials and Methods and its identity was confirmed using mass spectrometry experiments with the purity estimated to be around 98%. Analytical ultracentrifugation sedimentation equilibrium analysis (Fig. 3 Panel A) was performed on a fresh batch of protein, with no degradation products (Fig. 3 Panel B), after gel filtration chromatography and at 4 degrees Celsius to determine the oligomeric state of TLX LBD. Global fitting using mass conservation restraints of the protein distributions obtained at three separate rotor speeds, revealed a monomer-dimer equilibrium for TLX LBD self-association in solution. The best fit was obtained for a monomer-dimer equilibrium model with a Kd of 10 µM. 95% confidence interval limits for this dissociation constant were determined to be 4 µM<Kd<24 µM. Alternative models for a monomer-trimer or monomer-tetramer equilibrium were tested but gave rise to less optimal fitting of the raw data. These analytical ultracentrifugation results were confirmed by size-exclusion chromatography where TLX LBD elutes at a molecular weight corresponding to a dimer (Fig. 3 panel C).

### DSF Screening Identifies 190 Hits Defined as TLX Binders

**Figure 4:**
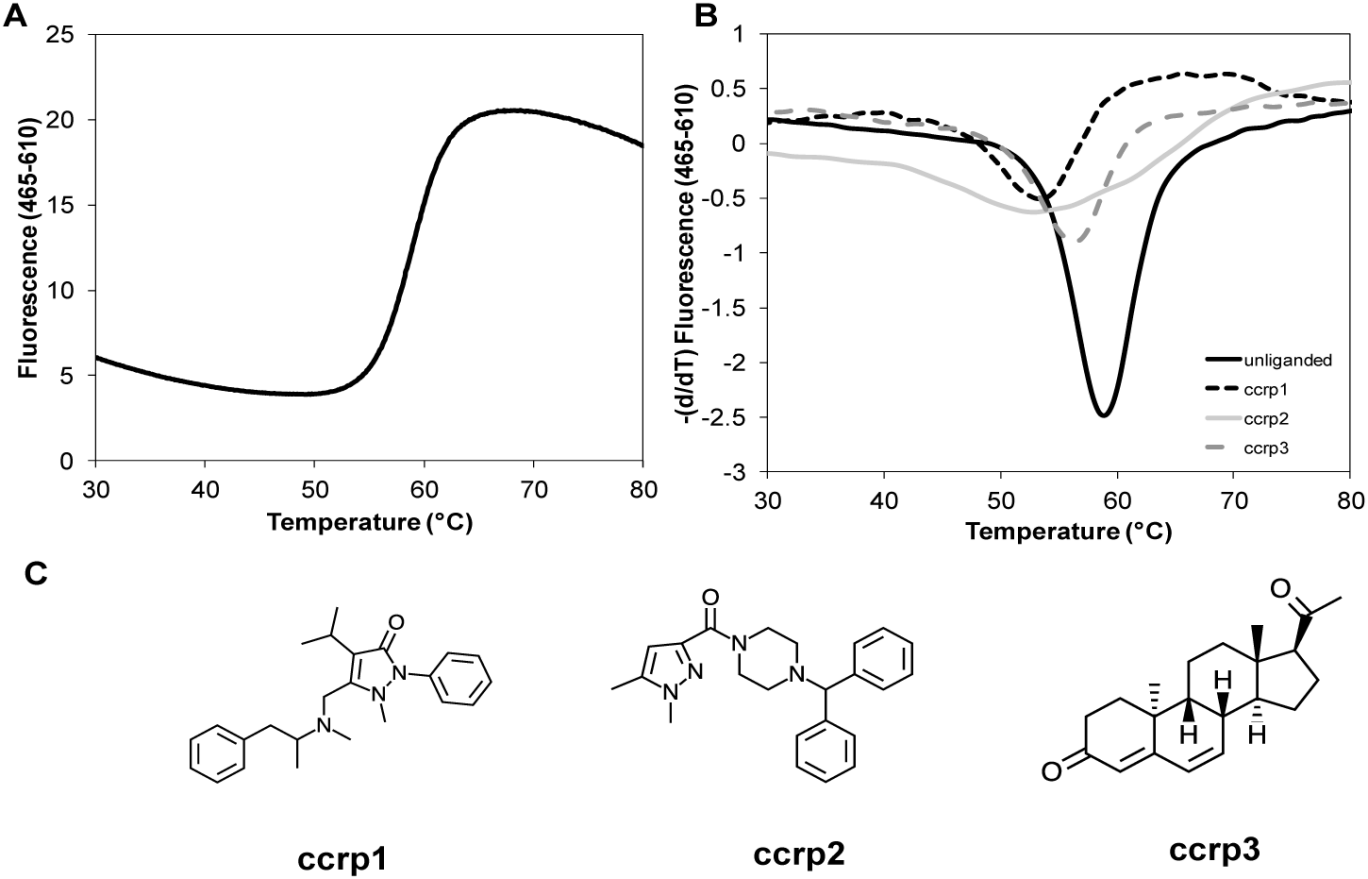
Results of direct binding assays using the DSF method. A. This panel represents the melting curve of purified TLX LBD in the presence of 5% DMSO. B. This panel allows a rapid visualization on how compounds binding to TLX LBD can shift the reference Tm. We plotted the first derivatives of the melting curves for unliganded TLX LBD and TLX LBD in presence of ccrp1, ccrp2 and ccrp3. While these curves were utilized to accurately calculate T_m_, a rough approximation of the Tm would be the minimum of the first derivative curves. In presence of the three compounds this minimum shifted downward. C. This panel displays the chemical structures of ccrp1, ccrp2, and ccrp3.

We analyzed binding of approximately 20,000 compounds to the purified recombinant TLX LBD using the DSF method. Briefly, this approach measures ligand-dependent changes in protein melting temperature (T_m_) via changes in presentation of buried hydrophobic surfaces [41]–[43]. Compounds came from in-house libraries comprised of NIH Clinical Collections I and II (450 and 281 molecules respectively that have a history of use in clinical trials), Prestwick Chemical Library (1200 approved drugs), Selleck Chemicals (a unique collection of 853 FDA approved drugs), NCI Diversity set (2,000 synthetic small molecules selected from the full NCI Screening Collection), NCI Mechanistic Set (879 compounds), NCI Natural Products (120 products derived from plants and microbes) and Maybridge/Thermo-Scientific HitFinder library (14,400 compounds selected to be non-reactive). In the presence of 5% DMSO (solvent control), TLX LBD has a very typical and well-described melting curve (Fig. 4 panel A). Indeed, the low temperature part of the curve has low initial fluorescence values and the slope of the curve approaches horizontal. As the protein begins to unfold with increasing temperature, exposure of hydrophobic residues results in a fluorescence increase, yielding a sigmoidal shape curve. A clear transition at 58.8±0.3°C (reference T_m_) is then observed until a plateau is reached which corresponds to completely unfolded protein.

We decided to select molecules that were able to change TLX stability by shifting the T_m_ by 0.9°C (3 times the SD for reference T_m_) or more. A total of 365 hits (hit rate of 1.8%) fell into this category (Table S1) and included ccrp1, ccrp2, and ccrp3 that displayed melting temperatures of 53.04±0.3°C; 52.49±1.1°C; 55.78±0.3°C, respectively (Table 1, Figure 4 Panels B and C).

**Table 1:** Description of the TLX ligands.

Name	Common names or IUPAC names	Manufacturer ID	Molecular Weight (Da)
ccrp1	Famprofazone	Santa Cruz Biotechnology sc-235122	377.52
ccrp2	1-(1,5-dimethylpyrazole-3-carbonyl)-4-(diphenylmethyl)piperazine	Maybridge SCR00686	374.48
ccrp3	Dydrogesterone	Santa Cruz Biotechnology sc-214952	312.45

Manual chemical inspections of substructures of the 365 initial hits were performed to remove Pan Assay Interference Compounds [44] (including reactive compounds, known aggregators, redox-active molecules) or compounds that could not fit inside the predicted TLX LBP based on size, volume and polar/hydrophobic features (Table S1). After this step, only 190 hits (final hit rate of 0.95%) were selected (Table S1). No non-specific interactions between the compounds and the fluorescent dye were detected. Notably, all 190 hits shifted the receptor T_m_ downward, indicating that the TLX LBD is destabilized upon binding of these compounds. This is opposite to the case for most other nuclear hormone receptors [42], in which ligand binding triggers an upward shift in T_m_ values that is concomitant with increased stability of the domain.

#### Three hits ccrp1, ccrp2 and ccrp3 are confirmed specific binders for TLX LBD

**Figure 5:**
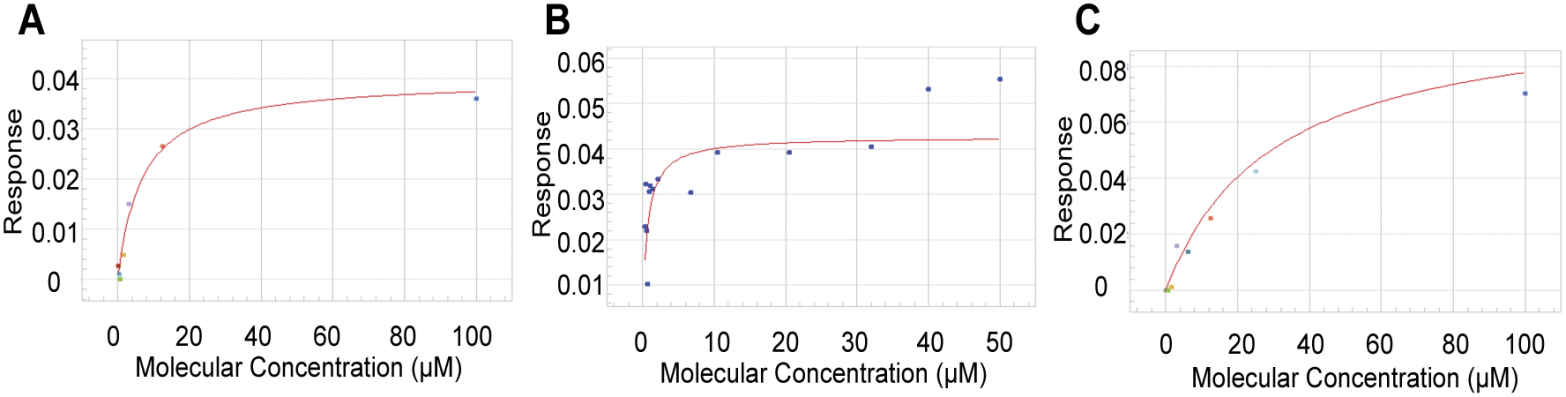
Results of direct binding assays for ccrp1, ccrp2 and ccrp3 on the TLX LBD using the Octet RED 384 instrument. A, B, C. Those panels represent the plotted steady-state response levels and the fitted binding isotherms. The purified TLX protein was immobilized onto the surfaces of Super-Streptavidin biosensors. Solutions of compounds ccrp1 (panel A), ccrp2 (panel B), ccrp3 (panel C) at 0.4–100 µM concentrations were tested against immobilized TLX LBD and reference surfaces composed of blocked biotinylated Streptavidin.

To verify DSF hits observed in primary screens and to determine if the interactions were truly stoichiometric, we performed an orthogonal direct binding assay. This approach relied upon the Octet Red 384 (FortéBio), an optical biosensor, to determine the binding kinetics for small molecules in a 384-well plate format. For this analysis, purified avi-tagged TLX LBD protein was immobilized onto the surface of Super-Streptavidin sensors. For reference surfaces, blocked biotinylated Streptavidin was immobilized onto another set of Super-Streptavidin sensors. Biosensors coated with TLX LBD and blocked biotinylated Streptavidin, were dipped separately into solutions of each of the putative 190 hits at a final concentration of 100 µM. This method confirmed binding of 24/190 compounds. Compounds defined as not binding to TLX LBD in this assay were removed from further considerations. Binding of 24 compounds was then evaluated at concentrations ranging from 100 µM to 400 nM. This revealed that three compounds (ccrp1, ccrp2 and ccrp3) produced a steady-state response in a dose-dependent manner (Fig. 5). Since purified avi-tagged TLX protein has a molecular weight of 27,000 Da and a loading signal of 10 nm; the maximum binding response for compounds with molecular weights around 300 Da would be 0.08–0.1 nm in these experiments. A maximum binding response above 0.1 would signify that the compounds bind with a super-stoichiometry. As expected, we reached 0.06–0.1 nm for the maximum response plateau of ccrp2 and ccrp3. At the highest concentrations tested, ccrp1 only attained a maximum binding response of 0.04 nm, likely due to a less effective loading of the protein onto the sensors. The equilibrium dissociation constant (K_d_) was determined using steady state analysis of binding affinities, assuming 1∶1 ligand-protein stoichiometry. Curve-fitting analysis of the corrected response isotherm estimated K_d_ values of 6.6±0.07 µM for ccrp1, 650±100 nM for ccrp2, and 27.5±3.5 µM for ccrp3.

#### Ccrp1, ccrp2 and ccrp3 modulate TLX transcriptional activity

**Figure 6:**
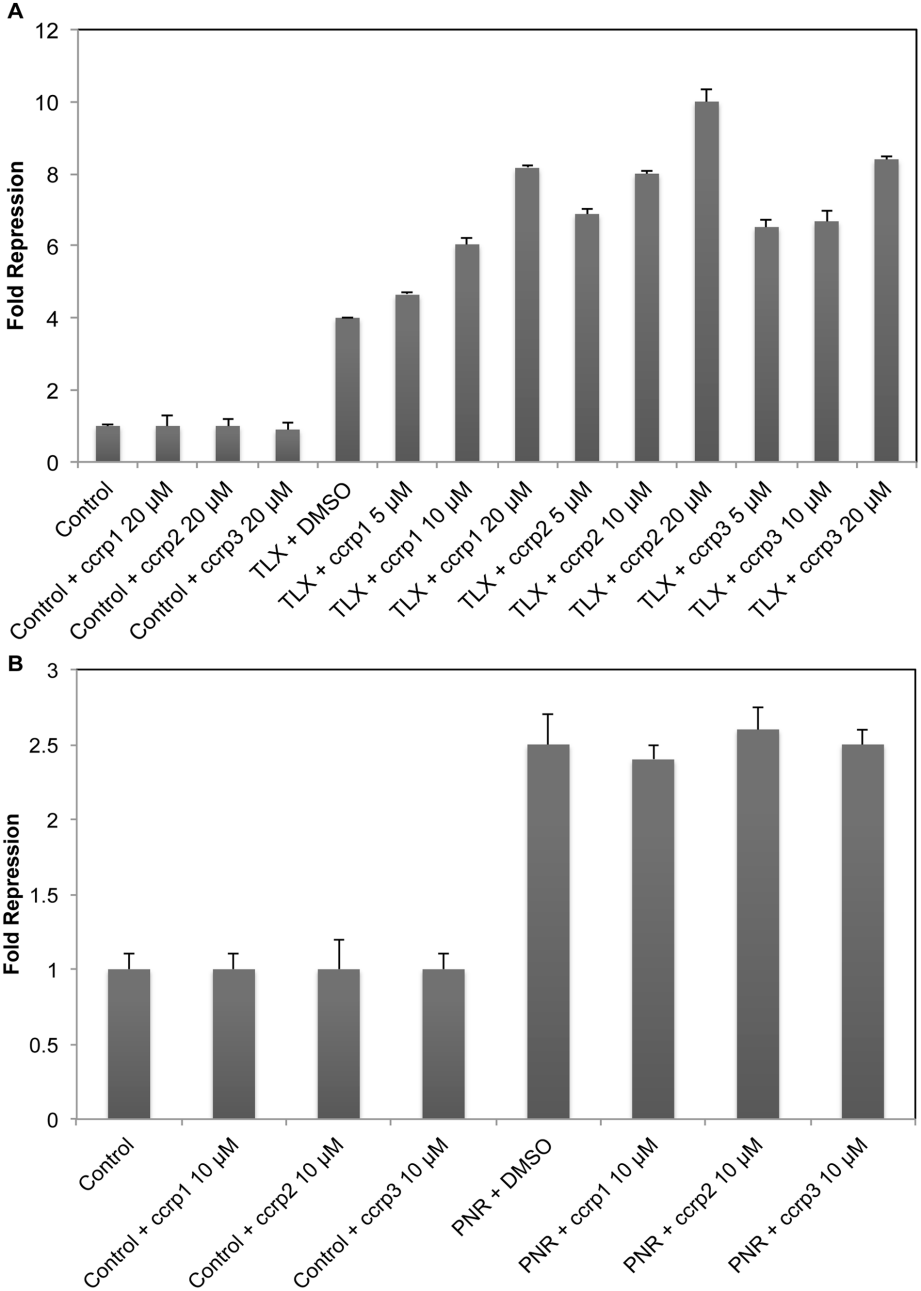
Modulation of TLX and PNR transcriptional activities by ccrp1, ccrp2 and ccrp3. A. Transfections of TLX LBD repress the UAS promoter leading to a decrease in luciferase activities compared to the control (cells transfected with empty GAL4 vector). Compounds ccrp1, ccrp2 and ccrp3 respectively enhance repressive transcriptional activity of TLX only in cells transfected with TLX LBD. HeLa cells transiently transfected with TLX LBD or empty GAL4 vector and the luciferase reporter gene were treated with either DMSO (0.1%, solvent control) or compounds of interest at different concentrations (indicated). Following 16 h treatments, luciferase activities were recorded and normalized. For each concentration point, data are shown as fold repression relative to control (cells transfected with empty GAL4 vector and treated with 0.1% DMSO), as average of three independent measurements, with experimental errors shown as black lines. B. HeLa cells transiently transfected with PNR LBD or empty GAL4 vector and the luciferase reporter gene were treated with DMSO (0.1%, solvent control) or ccrp1, ccrp2 and ccrp3 at 10 µM. Following 24 h treatments, luciferase activities were recorded and normalized. For each concentration point, data are shown as fold repression relative to control (cells transfected with empty GAL4 vector and treated with 0.1% DMSO), as average of three independent measurements, with experimental errors shown as black lines.

To verify that selected ligands modulate TLX activity, we assessed transcriptional activity of the TLX LBD in the absence and the presence of varying concentrations of ccrp1, ccrp2, and ccrp3. As predicted, binding of TLX LBD repressed a GAL4 UAS-dependent promoter (in the pGL4.35 reporter gene vector) (Fig. 6 panel A). This agrees with suggestions that TLX is a constitutive transcriptional repressor [6], [45]. In presence of the compounds of interest, we observed further repression of the GAL4-dependent promoter in the presence of GAL4-TLX LBD compared to DMSO-treated cells. Defined EC_50_ values for each compound were 9.2±1.0 µM for ccrp1, 1.0±0.3 µM for ccrp2, and 250±100 nM for ccrp3 (Fig. 6 panel A and Fig. S2). No analogous effects were seen in cells that were transfected with GAL4 DBD vector (Fig. S3). Thus, we conclude that hit TLX compounds potentiate TLX transcriptional repressive activity.

### Hit Compounds are not Promiscuous Ligands

**Figure 7:**
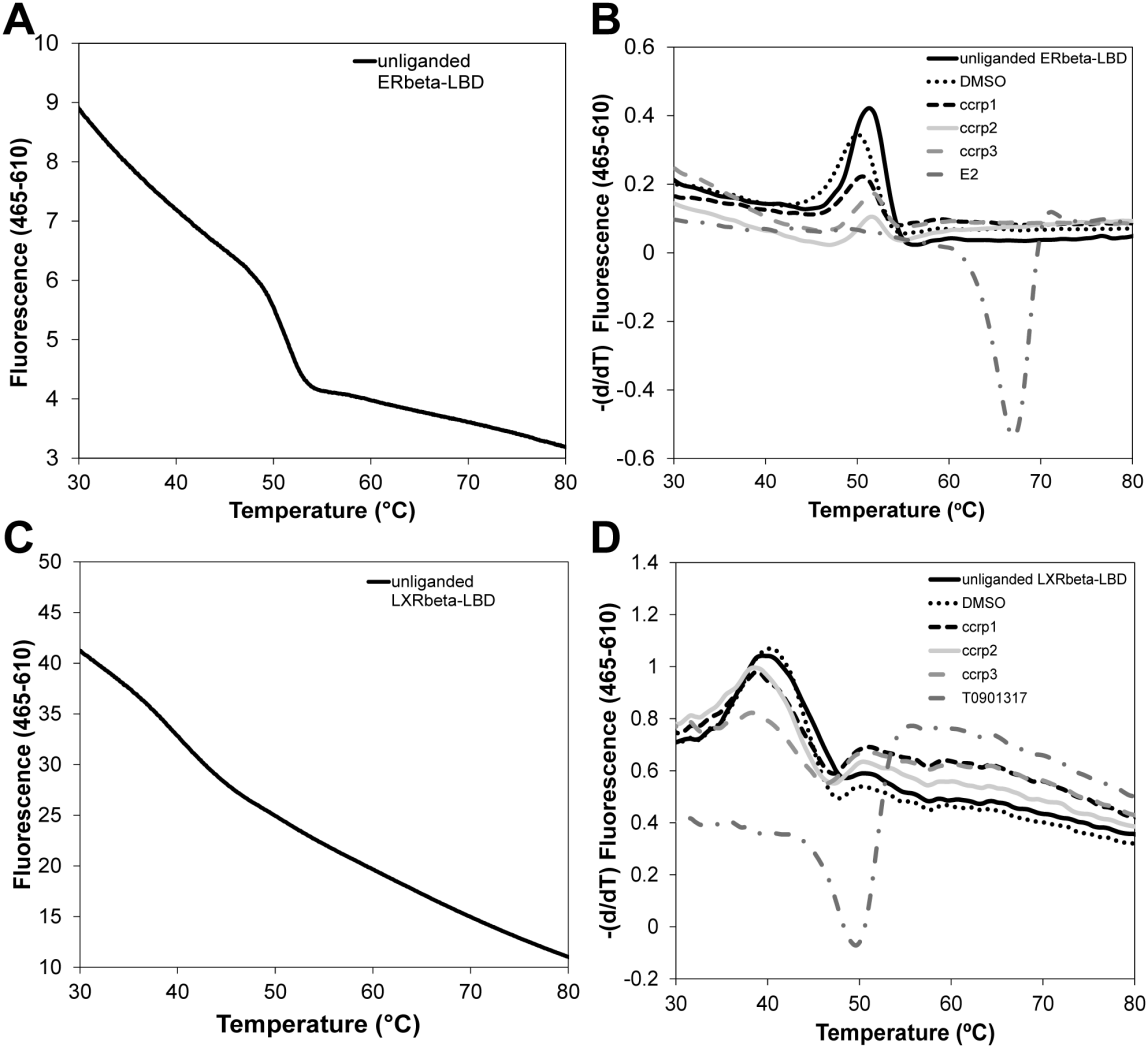
Specificity of ccp1, ccrp2 and ccrp3 towards unrelated nuclear receptors. A. This panel represents the melting curve of purified ERβ LBD in the absence of ligand. B. Melting temperature shifts for ERβ LBD in absence of compounds or treated with estradiol (E2), ccrp1, ccrp2 and ccrp3 at a final concentration of 500 µM. Neither compound demonstrates any significant effect on the melting temperatures compared to the shift induced by estradiol. C. This panel represents the melting curve of purified LXRβ LBD in the absence of ligand. D. Melting temperature shifts for LXRβ LBD in absence of compounds or treated with T0901317, ccrp1, ccrp2 and ccrp3 at a final concentration of 500 µM. Neither compound demonstrates any significant effect on the melting temperatures compared to the shift induced by T0901317.

We examined whether ccrp1, ccrp2, and ccrp3 could bind two unrelated nuclear receptors ERβ and LXRβ (Fig. 7). For accurate comparisons, we employed analogous DSF methods to that employed for TLX in absence or presence of compounds of interest (500 µM) or DMSO (5%, solvent control). Human ERβ LBD produced a denaturation curve with a melting point (T_m_) of ∼51**°**C (Fig. 7 Panel A) and LXRβ LBD produced a denaturation curve with a melting point (T_m_) of ∼39**°**C (Fig. 7 Panel C). Unliganded ERβ and LXRβ seem to behave like molten globular proteins with high initial fluorescence values at low temperatures, indicative of exposed hydrophobic residues that are accessible to Sypro Orange dye. Additionally, as the temperature increases, thermal energy decreases the affinity of the dye for exposed hydrophobic residues of the protein and the fluorescence is decreasing, resulting in a downward slope in the initial part of the melting curve.

The presence of reference compounds for both proteins dramatically changed the respective melting temperatures (Fig. 7 Panels B and D). For both proteins, the T_m_ in presence of E2 or T0901317 shifted upward implying a stabilization of the proteins upon binding. However, no significant shifts in T_m_ were recorded with either NR or any TLX interacting compound.

**Figure 8:**
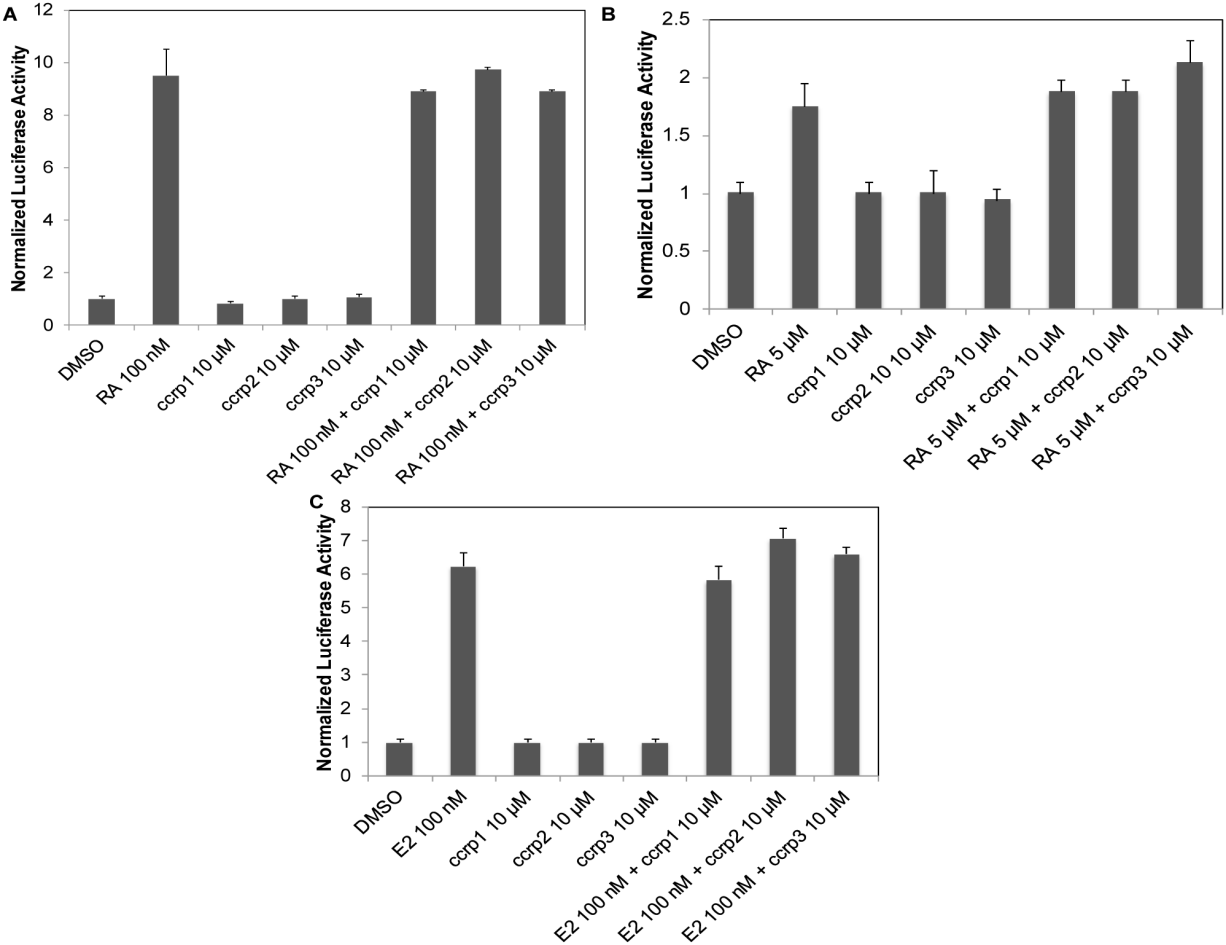
Modulation of RXRα, COUP-TFII and ERβ transcriptional activities by ccrp1, ccrp2 and ccrp3. A, B. HeLa cells transiently transfected with RXRα LBD (panel A) or COUP-TFII LBD (panel B) and the luciferase reporter gene were treated with either DMSO (0.1%, solvent control) or compounds of interest at 10 µM in absence or presence of 9-cis retinoic acid (RA) at different concentrations (100 nM for RXRα and 5 µM for COUP-TFII). C. HeLa cells transiently transfected with ERβ LBD and the luciferase reporter gene were treated with either DMSO (0.1%, solvent control) or ccrp1; ccrp2 and ccrp3 at 10 µM in absence or presence of E2 at 100 nM. For all panels, following 24 h treatments, luciferase activities were recorded and normalized. For each concentration point, data are shown relative to control (0.1% DMSO), as average of three independent measurements, with experimental errors shown as black lines.

We also tested ccrp1, ccrp2, and ccrp3 for their ability to modulate the transcriptional activity of several human NR LBDs using a luciferase reporter assay. Because of the high sequence identity between TLX and PNR, we investigated if the three TLX ligands we identified could modulate PNR transcriptional activity. None of the compounds affected PNR transcriptional activity (Fig. 6 Panel B). Because our homology model of TLX LBP was mainly designed using RXRα as a template and COUP-TFII displays high sequence identity with TLX, we decided to investigate the ability of ccrp1, ccrp2, and ccrp3 to modulate the transcriptional activity of both receptors. No effect on luciferase activity was detected when the compounds were tested alone or in competition with the 9-cis retinoic acid, a potent agonist for RXRα and COUP-TFII (Fig. 8 Panels A and B). We also tested in transactivation assays our compounds of interest on a steroid hormone receptor, ERβ. No effect on luciferase activity was detected when the compounds were tested alone or in competition with E2, a potent agonist (Fig. 8 Panel C). This experiment also confirmed our DSF findings: no binding was observed between ccrp1, ccrp2, or ccrp3 and the purified recombinant ERβ LBD protein. Based on these data, we conclude that the identified ligands bind preferentially to TLX.

## Discussion

It is important to identify TLX ligands to dissect TLX dependent regulatory pathways. Presently, studies of TLX function have been restricted to mouse knockout models or siRNA knockdown approaches [4], [5], [8], [11], [12], [14]–[16], [19], [20]. These studies revealed striking roles for TLX in NSC proliferation and renewal, neural differentiation, and the emergence of glioma. However, new ligands, which regulate TLX would permit us to determine how modulation of TLX activity alters expression of key target genes in real time and to monitor the effects of changes in TLX activity in cell backgrounds and animal models with normal levels of TLX expression. There are interesting suggested applications for small molecule therapeutics which target TLX [17]. Because of its role in neurogenesis, small molecules that bind TLX could be used as complementary therapeutic approaches to increase the size of the NSC population or trigger NSC differentiation to reverse damage caused by neurodegenerative diseases [17]. Because of its role in glioma, small molecules that bind TLX could also be useful in NSC-derived brain tumors [20]. In either case, TLX ligands are unlikely to display side effects in other tissues because TLX expression is limited to the brain.

At the start of our study, it was not clear whether TLX harbors a LBP or whether the putative LBP is filled, as seen with hydrophobic substituents that fill the LBP of Nurr1 [40] and render the latter receptor a true orphan devoid of endogenous and exogenous ligands. Our initial homology modeling study suggests that TLX belongs to an emerging subgroup of NRs including PNR, SHP, and DAX, which lack helices α1 and α2. The crystal structures of the apo-forms of PNR, DAX, and SHP LBDs, along with the close TLX homolog COUP-TFII, reveal an auto-repressed conformation with two main features. First, a kink between helices α10 and α11 closes the entrance of the LBP. Second, helix α12 binds into the AF-2 site, mimicking the binding of a coregulator and thus preventing coactivator recruitment. It is also noteworthy, however, that PNR, which is homologous to TLX and lacks helices α1 and α2, and COUP-TFII, which also displays high sequence identity with TLX, can both bind ligands. Our modeling studies, based on a transcriptionally active conformation of the closely related RXRα suggest that TLX may indeed form an accessible, albeit unusual, LBP that would be partly buried within the hydrophobic core of the protein and partly solvent exposed and should therefore be amenable to small molecule drug development.

Recombinant TLX LBD displayed the capacity to homodimerize in solution. This was somewhat surprising, given that previous studies have suggested that full length TLX acts as an obligate monomer [14]. While we suspect that this observation may mean that TLX could exhibit the capacity to act as a dimer in some contexts, and TLX homologs such as COUP-TFII and PNR can dimerize [28], [37], we emphasize that our experiments were performed with TLX LBD and not full length TLX. Thus, the physiologic significance of this observation must remain questionable at present.

Initial screens to identify TLX binders used recombinant human TLX LBD in direct binding studies. Our primary screen was a DSF approach, which assesses ligand-dependent changes in protein thermostability. DSF is rapid, inexpensive, and requires small amounts of protein but can generate large numbers of false positives [41]. After removing compounds that frequently interfere with biochemical assays (PAINS) [44], we detected 190 possible hits, which were verified using a two-step biolayer interferometry approach (with the Octet Red 384 instrument). The 190 hits were initially tested at a single concentration (100 µM), which revealed a large false positive rate and eliminated 166 compounds. The remaining 24 ligands were then tested in dose-response curves. Three compounds ccrp1, ccrp2, and ccrp3, displayed quick association and dissociation and reached a response plateau at the highest concentrations tested. Other compounds that initially scored as hits failed to show response saturation at the highest concentrations indicating weak binding with TLX LBD or displayed slow association and/or slow dissociation. The latter phenomenon may be associated with compounds containing reactive or chelating groups that bind irreversibly to the target and with super-stoichiometric compounds, which can exhibit slow association and/or slow dissociation due to binding to proteins in the form of micelles or other aggregates. These characteristics are not bad *per se* but such hits need to be validated with an experimental design that can establish the mechanism of interaction. For this study, we therefore decided to pursue compounds exemplified by ccrp1, ccrp2 and ccrp3, which displayed typical “square-pulse sensorgrams” with clear kinetic pattern, fast association and dissociation. The dramatic decrease in the amount of hits from 365 down to three verified compounds reinforces the notion that it is important to use a combination of direct binding assays early on during the screening process.

Notably, all the hits identified by DSF shifted the TLX melting temperature downward suggesting that the protein is destabilized upon ligand binding. There are three possible explanations for this phenomenon. First, TLX LBP could have trapped an endogenous ligand from *E. coli* during expression/purification, and the downward shift could be due to the displacement of this ligand. This downward shift has also been shown with the Liver Receptor Homolog 1 (LRH-1) [43]. However, preliminary mass spectrometry analysis based on the estimation of a possible molecular weight difference between folded and unfolded TLX LBD proteins seems to discard that hypothesis (data not shown). Second, addition of compounds could change the oligomeric state of the protein. However, analytical ultracentrifugation in the presence of the three hit compounds at 100 µM showed no effect on the dissociation constant for dimerization, ruling out the possibility that the compounds are acting as denaturants or altering monomer/dimer equilibrium (data not shown). We favor another idea. As mentioned above, X-ray structures of the closely related unliganded COUP-TFII [28], SHP [39], and PNR [37] reveal a stable auto-repressed condition, with helix α11 closing the entrance of the LBP and the helix α12 folded in a position that blocks coactivator binding. We expect that the LBP would have to expand to accommodate ligands, and that this would change the receptor LBD from a tightly folded auto-repressed conformation to a more open organization. Ligands binding to the stable auto-repressed conformation are likely to destabilize it eventually promoting the release of Helix α12 from the AF-2 site. Because of strong homologies between TLX, COUP-TFs, and PNR, we suggest that this idea explains destabilization of the TLX LBD upon ligand binding.

We emphasize that even the liganded “destabilized” form of the TLX LBD is quite stable compared to other liganded nuclear receptor LBDs suggesting that it could remain functional and explaining continued transcriptional repressive activity in the presence of ligands. Confirmed TLX binders, ccrp1, ccrp2, and ccrp3, potentiate TLX transrepressive activity in transfections that utilized a GAL4-LBD fusion protein [6], [45]. None of the three compounds displayed toxicity or apparent TLX-independent effects when tested at different concentrations. Thus, all three compounds could constitute a good starting point to derive and discover new families of ligands for TLX. Ccrp1 (famprofazone) is a non-steroidal anti-inflammatory agent of the pyrazolone series, for whom toxicity in humans has already been studied. The structure of ccrp2 is similar to ccrp1 with pyrazole and piperazine functional groups and commercial derivatives of this compound can be purchased. Ccrp3 (dydrogesterone also known as 9β,10α-pregna-4,6-diene-3,20-dione) is a steroidal progestin meaning that other steroids should be tested in the future on TLX. Due to their chemical structures and because compounds with steroidal scaffolds have been found to bind inside other NR LBPs, we strongly believe that these compounds bind inside TLX LBP.

While these compounds or subsequent derivatives could be useful tools to study mechanisms of TLX action at target genes, significant improvements would be needed to generate preliminary TLX drugs. First, all initial hits are relatively low potency ligands, and it will be important to elaborate upon these compounds to generate specific ligands that bind TLX with higher affinity. This may require improved understanding of the binding mode of the ligand for informed structure activity relationship profiling. Second, all of our ligands potentiate TLX actions. Since it is likely to become important to reverse TLX repressive activity to trigger NSC differentiation in neurodegenerative diseases and block TLX activity in glioma, it will be important to understand the structural basis for TLX-dependent repressive activities to rationally generate ligands to block these mechanisms. The fact that we have been able to identify ligands that directly bind to TLX with affinities in the high nanomolar to low micromolar range and enhance its repressive activity proves that TLX is druggable. The question of whether it will be possible to generate high affinity ligands that can reverse TLX repressive activities remains open.

## Supporting Information

Figure S1 **Ramachandran Plot of the TLX LBD model.** This plot represents the torsional angles of the residues in the polypeptide chains of TLX LBD. 186 residues have their torsional angles in preferred regions, 9 residues in the allowed regions and only 2 residues have not allowed torsional angles.
(TIF)

Figure S2 **Dose-responses curves for ccrp1, ccrp2 and ccrp3.** A. Transfections of TLX LBD repress the UAS promoter leading to a decrease in luciferase activities. B, C and D. HeLa Cells transiently transfected with TLX LBD and the luciferase reporter gene were treated with either DMSO (0.1%, solvent control) or compounds of interest at different concentrations (indicated). Following 16 h treatments, luciferase activities were recorded and normalized. For each concentration point, data are shown as fold repression relative to cells transfected with TLX LBD and treated with 0.1% DMSO, as average of three independent measurements, with experimental errors shown as black lines. EC_50_s have been calculated using Prism 6 (GraphPad).
(TIF)

Figure S3 **Results of transactivation assays in absence of TLX.** A, B and C. Compounds ccrp1, ccrp2 and ccrp3 respectively do not affect the luciferase activities in absence of TLX. HeLa cells transiently transfected with empty GAL4 vector and the luciferase reporter gene were treated with either DMSO (0.1%, solvent control) or compounds of interest at different concentrations (indicated). Following 16 h treatments, luciferase activities were recorded and normalized. For each concentration point, data are shown relative to control (cells transfected with empty GAL4 vector and 0.1% DMSO), as average of three independent measurements, with experimental errors shown as black lines.
(TIF)

Table S1 **List of compounds selected as hits after the DSF experiments.** This table summarizes for each library, the compounds names or if unavailable the IUPAC names for all the compounds able to bind to TLX LBD after the DSF experiments. For each library, we separated these compounds in two categories: those that we eliminated because they were identified as PAINS or because their chemical structures were unlikely to fit TLX LBP, and those that we decided to keep and further characterized using Biolayer Interferometry. However, in the case of the Maybridge Library, we kept all the compounds since Maybridge Library is defined as a “clean” library without any reactive compounds.
(PDF)
