# Supplementary figures and images for: The Human Orphan Nuclear Receptor Tailless (TLX, NR2E1) Is Druggable
Source: PLoS One. 2014 Jun 17;9(6):e99440. doi: 10.1371/journal.pone.0099440 (PMC4060991; doi:10.1371/journal.pone.0099440)

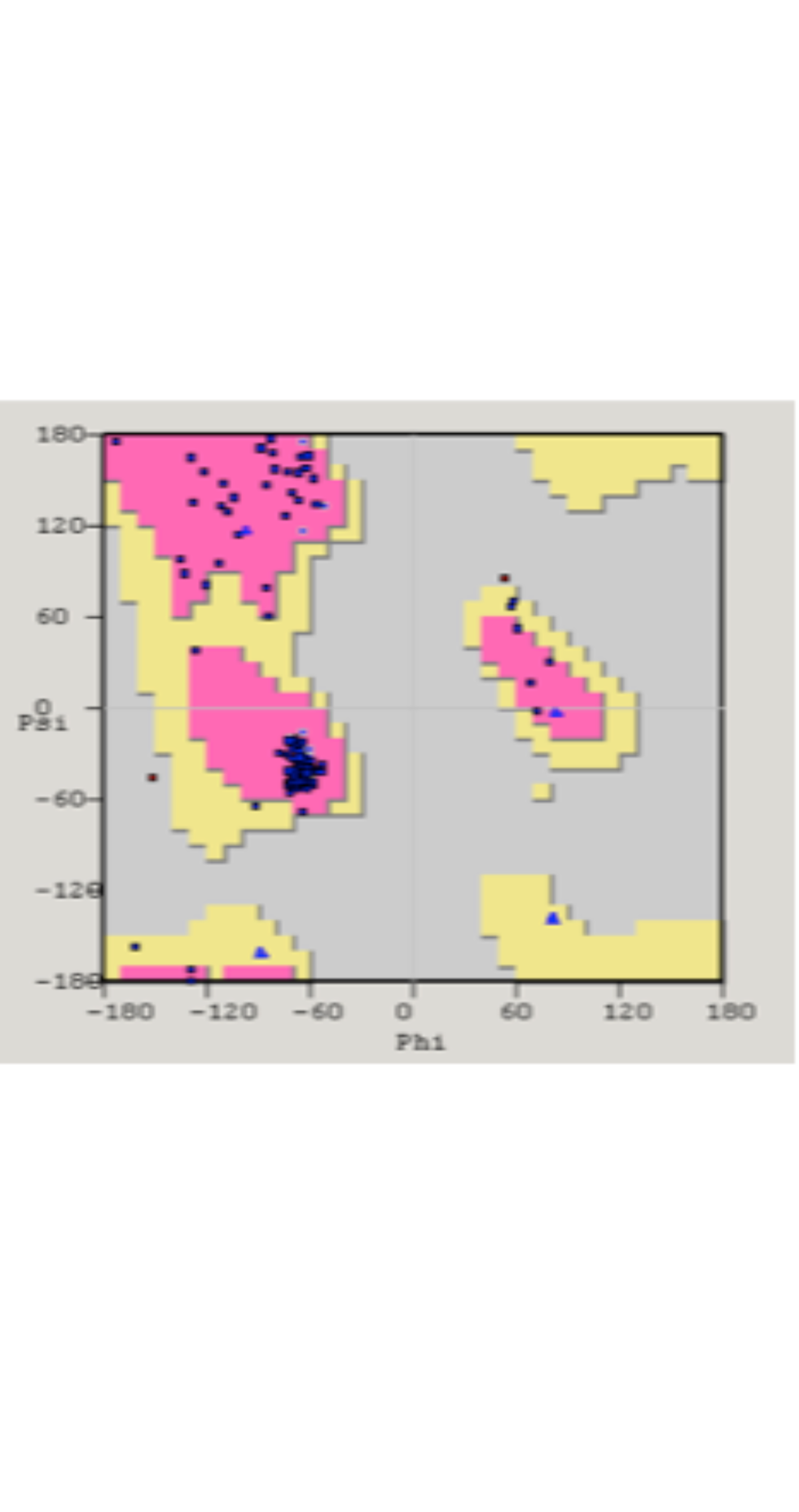

Supplement: Figure S1 — Ramachandran Plot of the TLX LBD model. This plot represents the torsional angles of the residues in the polypeptide chains of TLX LBD. 186 residues have their torsional angles in preferred regions, 9 residues in the allowed regions and only 2 residues have not allowed torsional angles. (TIF) [file pone.0099440.s001.tif]

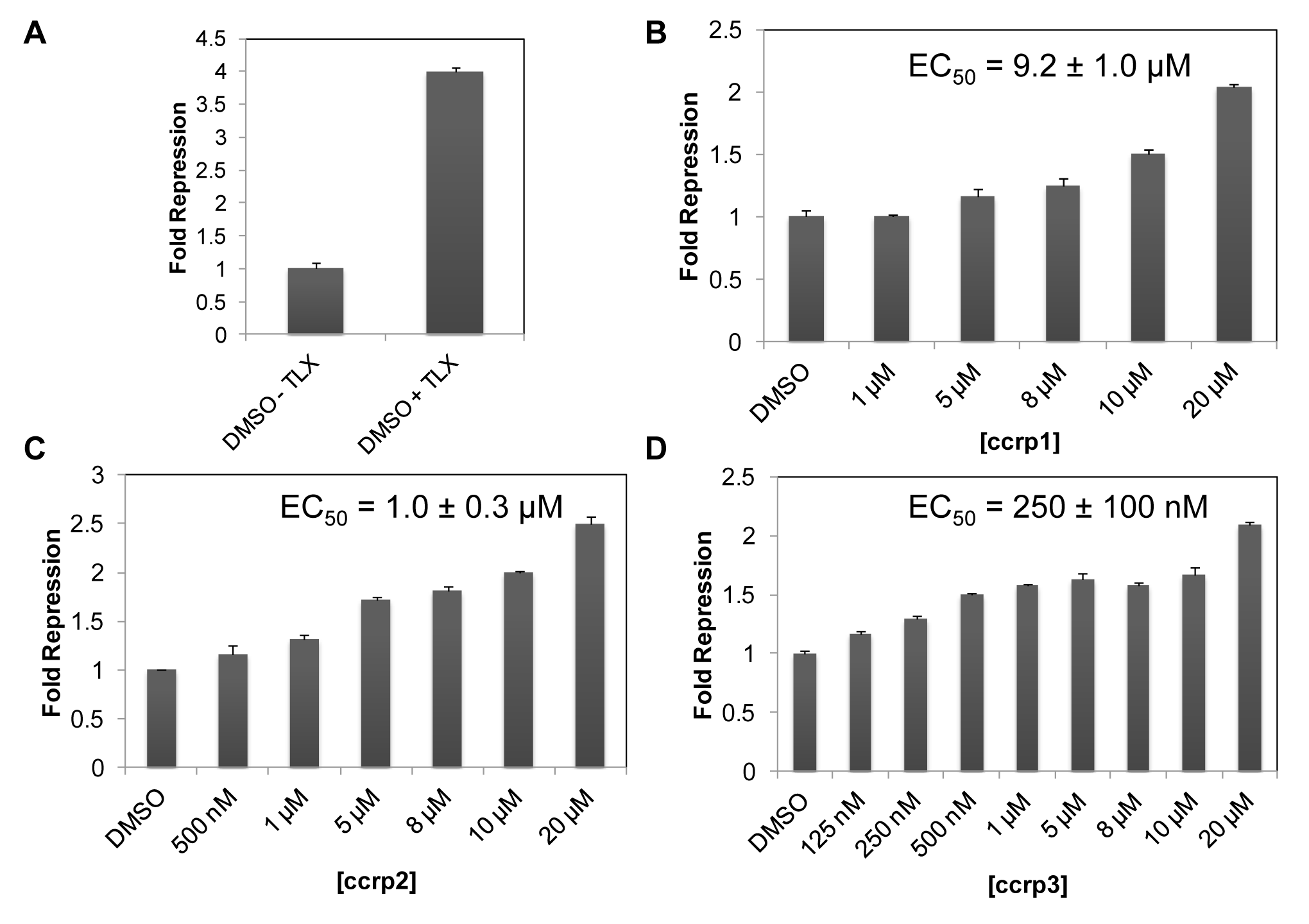

Supplement: Figure S2 — Dose-responses curves for ccrp1, ccrp2 and ccrp3. A. Transfections of TLX LBD repress the UAS promoter leading to a decrease in luciferase activities. B, C and D. HeLa Cells transiently transfected with TLX LBD and the luciferase reporter gene were treated with either DMSO (0.1%, solvent control) or compounds of interest at different concentrations (indicated). Following 16 h treatments, luciferase activities were recorded and normalized. For each concentration point, data are shown as fold repression relative to cells transfected with TLX LBD and treated with 0.1% DMSO, as average of three independent measurements, with experimental errors shown as black lines. EC50s have been calculated using Prism 6 (GraphPad). (TIF) [file pone.0099440.s002.tif]

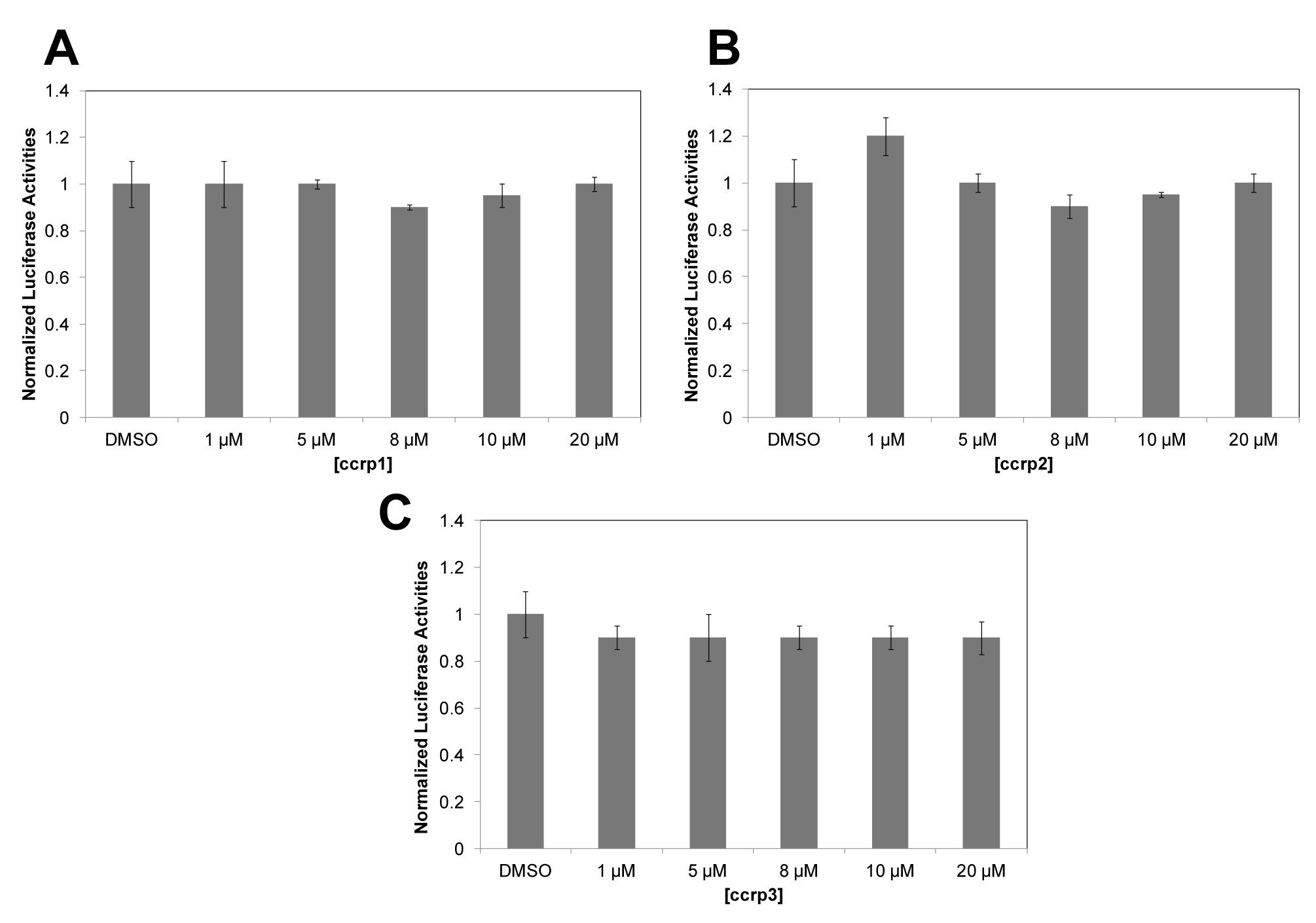

Supplement: Figure S3 — Results of transactivation assays in absence of TLX. A, B and C. Compounds ccrp1, ccrp2 and ccrp3 respectively do not affect the luciferase activities in absence of TLX. HeLa cells transiently transfected with empty GAL4 vector and the luciferase reporter gene were treated with either DMSO (0.1%, solvent control) or compounds of interest at different concentrations (indicated). Following 16 h treatments, luciferase activities were recorded and normalized. For each concentration point, data are shown relative to control (cells transfected with empty GAL4 vector and 0.1% DMSO), as average of three independent measurements, with experimental errors shown as black lines. (TIF) [file pone.0099440.s003.tif]
