# Supplementary material for: The Human Orphan Nuclear Receptor Tailless (TLX, NR2E1) Is Druggable
Source: PLoS One. 2014 Jun 17;9(6):e99440. doi: 10.1371/journal.pone.0099440 (PMC4060991; doi:10.1371/journal.pone.0099440)
Supplement: Table S1 — List of compounds selected as hits after the DSF experiments. This table summarizes for each library, the compounds names or if unavailable the IUPAC names for all the compounds able to bind to TLX LBD after the DSF experiments. For each library, we separated these compounds in two categories: those that we eliminated because they were identified as PAINS or because their chemical structures were unlikely to fit TLX LBP, and those that we decided to keep and further characterized using Biolayer Interferometry. However, in the case of the Maybridge Library, we kept all the compounds since Maybridge Library is defined as a “clean” library without any reactive compounds. (PDF) [file pone.0099440.s004.pdf]

Supporting Information Table S1

NIH Clinical Collections (I & II)

**Hits from DSF not tested in Biolayer Interferometry**	**Hits from DSF tested in Biolayer Interferometry**
Pizotyline	Clotrimazole
Loxapine	Indatraline
Loratadine	Ethynylestradiol
Diphenylcyclopropenone	Benzeneacetic acid, 2-[(2,6-dichlorophenyl)amino]-, monosodium salt
Paroxetine	Rosiglitazone maleate
Maprotiline hydrochloride	Benproperine phosphate
Trimebutine maleate	Esomeprazole Mg
Lofepramine	Sertraline
Midazolam hydrochloride	Pantoprazole sodium
Olanzapine	Torasemide
Moxifloxacin hydrochloride	Valdecoxib
Cilastatin sodium	Rosiglitazone HCl
Voriconazole	Beclomethasone
Tadalafil	Corticosterone
Vinorelbine tartrate	Norflex
Palonosetron hydrochloride	Benzhexol hydrochloride
1-benzylimidazole	19-Norethidrone acetate
Annoyltin	6Alpha-Methyl-11Betahydroxyprogesterone
Chlorpromazine	19-Norethindrone
Thioridazine	Procyclidine Hydrochloride
Acetamide, 2-amino-N-(1-methyl-1,2diphenylethyl)	Tamoxifen
Trifluoperazine	Meclomen
(7R)-7,14,16-trihydroxy-3-methy-3,4,5,6,7,8,9,10,11,12-decahydro-1H-2-benzoxacyclotetradecin-1-one	Testosterone
Nitrazepam	(R)-Fluoxetine Hydrochloride
Rolipram	Sertraline
Amitriptyline hydrochloride	Ketoconazole
Desipramine hydrochloride	6-alpha-hydrocortisol 21-acetate
3-(3-Chloro-5H-Dibenzo[B,F]Azepin-5-YL)-N,N-Dimethylpropan-1-Amine	

Prestwick Chemical Library

Hits from DSF not tested in Biolayer Interferometry	Hits from DSF tested in Biolayer Interferometry
Triflupromazine hydrochloride	Captopril
Benoxinate hydrochloride	Ticlopidine hydrochloride
Oxethazaine	Acetohexamide
Chlorpromazine hydrochloride	Minaprine dihydrochloride
Diphenhydramine hydrochloride	Dibucaine
Nicergoline	Hydralazine hydrochloride
Imipramine hydrochloride	Phenelzine sulfate
Amitryptiline hydrochloride	Flavoxate hydrochloride
Thioridazine hydrochloride	Iproniazide phosphate
Alverine citrate salt	Fendiline hydrochloride
Norethindrone	Labetalol hydrochloride
Nortriptyline hydrochloride	Mefexamide hydrochloride
Ethacrynic acid	Clemizole hydrochloride
Praziquantel	Glibenclamide
Catharanthine	Androsterone
Clomipramine hydrochloride	Amlodipine
Asenapine maleate	Bisacodyl
Quinidine hydrochloride monohydrate	Epiandrosterone
Homochlorcyclizine dihydrochloride	Benfluorex hydrochloride
Perhexiline maleate	Bepridil hydrochloride
Oxybutynin chloride	Fusidic acid sodium salt
Pimethixene maleate	Fentiazac
Clemastine fumarate	Papaverine hydrochloride
Mebeverine hydrochloride	Cilostazol
Trifluoperazine dihydrochloride	Fluoxetine hydrochloride
Clofilium tosylate	Gliclazide
Fluphenazine dihydrochloride	Prenylamine lactate
Testosterone propionate	Medrysone
Chlorprothixene hydrochloride	Flunixin meglumine
Suloctidil	**Dydrogesterone (ccrp3)**
Zotepine	Diethylstilbestrol
Dibenzepine hydrochloride	Estropipate
Corticosterone	Butamben
Cyclobenzaprine hydrochloride	3-alpha-hydroxy-5-beta-androstan-17-one
Dequalinium dichloride	**Famprofazone (ccrp1)**
Quetiapine	Finasteride
Prochlorperazine dimaleate	Cloperastine hydrochloride
Metixene hydrochloride	Isocarboxazid
Terconazole	Pyrithyldione
Azelastine hydrochloride	Fosfosal
Pentamidine isethionate	Butylparaben
Mirtazapine	Monobenzone

N-[4-[4-(2-methoxyphenyl)piperazin-1-yl]butyl]naphthalene-2-carboxamide dihydrochloride	Nylidrin
Monensin sodium salt<br>Tazobactam	Propofol<br>Deoxycorticosterone
Butirosin disulfate salt	Urosiol
Glimepiride	Canrenone
Suxibuzone	Piperidolate hydrochloride
Atractyloside potassium salt	Nandrolone
Adenosine 5'-(trihydrogen diphosphate), 5'-5'-ester with 3-(aminocarbonyl)-1-β-D-ribofuranosylpyridinium hydroxide, inner salt	Alfaxalone
Warfarin	Losartan
Pempidine tartrate	
Thimerosal	
Auranofin	
Suprofen	
Felbinac	
Oxalamine citrate salt	
Diloxanide furoate	
Iocetamic acid	
Nitrendipine	
Thioperamide maleate	
Quinapril hydrochloride	
Paroxetine hydrochloride	
Tolmetin sodium salt dehydrate	
S(-)Eticlopride hydrochloride	
Theobromine	
Loversol	
Rabeprazole Sodium salt	
Enilconazole	
Darifenacin hydrobromide	
Pivmecillinam hydrochloride	
Pizotifen malate	
Deptropine citrate	
Carprofen	
Vardenafil	
Ambrisentan	
Desloratadine	
Ibudilast	
Benztropine mesylate	
Bosentan	

Selleck Library

Hits from DSF not tested in Biolayer Interferometry	Hits from DSF tested in Biolayer Interferometry
Deforolimus	Masitinib
ZSTK474	Roscovitine (CYC202)
NVP-ADW742	TGX-221
SGX-523	Raloxifene
Everolimus (RAD001)	2-Methoxyestradiol
MLN8237	Cinacalcet hydrochloride
Danoprevir	Progesterone
Ketoconazole	Fluvastatin Sodium
Loratadine	Phloretin
Daptomycin	CYT387
Aurora A Inhibitor I	
Vinorelbine	
N5-(6-Aminohexyl)-3-isopropyl-N7-benzylpyrazolo[1,5-a]pyrimidine-5,7-diamine	
Clotrimazole	
Testosterone cypionate	
Loperamide hydrochloride	
OSI-027	
6,7-Dihydro-4-hydroxy-3-(2'-hydroxy[1,1'-biphenyl]-4-yl)-6-oxo-thieno[2,3-b]pyridine-5-carbonitrile	

NCI Mechanistic Set

Hits from DSF not tested in Biolayer Interferometry	Hits from DSF tested in Biolayer Interferometry
Dichlorocadmium	1-(2-chloroethyl)-1-nitroso-3-[(1-oxidopyridin-1-ium-3-yl)methyl]urea
[acetyl(methylcarbamoyl)amino] N-methylcarbamate	(E)-2-(benzoyl)-3-naphthalen-2-ylprop-2-enenitrile
2-hydroxytetradecanoic acid	2-(chloromethyl)-1,3-dinitro-5-(trifluoromethyl)benzene
Kauroic acid	2-(4-methylphenyl)sulfonyl-5-nitrofuran
1-[(2-chlorophenyl)-di(phenyl)methyl]imidazole	4-(morpholin-4-ylmethyl)-1-phenylnon-1-en-3-one
Chlorogold; Triethylphosphane	4-[[(8-hydroxyquinolin-7-yl)-phenylmethyl]amino]benzoic acid
Tributyl(chloro)plumbane	7,8,8a,9-tetrachloro-1,4a-dimethyl-7-propan-2-yl-2,3,4,4b,5,6,8,9,10,10a-decahydrophenanthrene-1-carboxylic acid
acetyloxy-(2,3,4,5,6-pentamethylphenyl)mercury	[(E,6R)-6-[(2S,8S,9R,10R,13R,14S,16R,17R)-2,16-dihydroxy-4,4,9,13,14-pentamethyl-3,11-dioxo-2,7,8,10,12,15,16,17-octahydro-1H-cyclopenta[a]phenanthren-17-yl]-6-hydroxy-2-methyl-5-oxohept-3-en-2-yl] acetate
2-[(1,3-dioxoinden-2-yl)-(4-nitrophenyl)methyl]indene-1,3-dione	
copper 3-(4-dimethylaminophenyl)prop-2-enoate	
Acetyloxymercury; Phenanthrene	
[acetyloxy-di(phenyl)plumbyl] acetate	
benzoyloxy-[[5-(benzoyloxymercuriomethyl)-1,4-dioxan-2-yl]methyl]mercury	
2-[6-[[2-[5-[5-[6-hydroxy-6-(hydroxymethyl)-3,5-dimethyloxan-2-yl]-3-methyloxolan-2-yl]-5-methyloxolan-2-yl]-9-methoxy-2,4,10-trimethyl-1,6-dioxaspiro[4.5]decan-7-yl]methyl]-3-methyloxan-2-yl]propanoic acid	
1-[10-(4-amino-2-methylquinolin-1-ium-1-yl)decyl]-2-methylquinolin-1-ium-4-amine chloride	
(2S)-N-[(2R)-1-[[(3S,6S,8S,12S,13R,16S,17R,20S,23S)-13-[(2S)-butan-2-yl]-12-hydroxy-20-[(4-methoxyphenyl)methyl]-6,17,21-trimethyl-3-(2-methylpropyl)-2,5,7,10,15,19,22-heptaoxo-8-propan-2-yl-9,18-dioxa-1,4,14,21-tetrazabicyclo[21.3.0]hexacosan-16-yl]amino]-4-methyl-1-oxopentan-2-yl]-1-[(2S)-2-hydroxypropanoyl]-N-methylpyrrolidine-2-carboxamide	

1-[4-[2-[4-[2-hydroxy-3-(2,4,4-trimethylpentan-2-ylamino)propoxy]phenyl]propan-2-yl]phenoxy]-3-(2,4,4-trimethylpentan-2-ylamino)propan-2-ol hydrochloride	
[5-hydroxy-6-[2-hydroxy-3-[[4-[[2-hydroxy-7-[3-hydroxy-5-methoxy-6,6-dimethyl-4-(5-methyl-1H-pyrrole-2-carbonyl)oxyoxan-2-yl]oxy-8-methyl-4-oxochromen-3-yl]carbamoyl]-3-methyl-1H-pyrrole-2-carbonyl]amino]-8-methyl-4-oxochromen-7-yl]oxy-3-methoxy-2,2-dimethyloxan-4-yl] 5-methyl-1H-pyrrole-2-carboxylate	
Antibiotic 382B	
N,N'-di(acridin-9-yl)octane-1,8-diamine	
Rapamycin	
N-[1-[[1-[[1-[[1-[[1-[[1-[[1-[1-[2-hydroxyethyl(methyl)amino]propan-2-ylamino]-2-methyl-1-oxopropan-2-yl]amino]-2-methyl-1-oxopropan-2-yl]amino]-1-oxopropan-2-yl]amino]-3-methyl-1-oxopentan-2-yl]amino]-2-methyl-1-oxopropan-2-yl]amino]-2-methyl-1-oxopropan-2-yl]amino]-1-oxopropan-2-yl]-4-methyl-1-[1-(2-methyldecanoyl)pyrrolidine-2-carbonyl]-6-(2-oxobutyl)piperidine-2-carboxamide	

NCI Diversity Set

Hits from DSF not tested in Biolayer Interferometry	Hits from DSF tested in Biolayer Interferometry
4-[(6-chloro-2-methoxyacridin-9-yl)amino]-5-methyl-2-propan-2-ylphenol	5,6-dichloro-2-[3-(trifluoromethyl)phenyl]-1H-imidazo[4,5-b]pyrazine
2-(4-hydroxy-3-methoxyphenyl)-6-(3-methylanilino)-4-oxo-2,3-dihydro-1,3-thiazine-5-carbonitrile	2-(4-chlorophenyl)-6-methoxyquinoline-4-carboxylic acid
Succinic acid	2-[(4-chloro-1-hydroxynaphthalene-2-carbonyl)amino]-2-methylpropanoic acid
(Z)-but-2-enedioic acid; 1-methyl-4-(8-methylsulfanyl-5,6-dihydrobenzo[b][1]benzothiepin-6-yl)piperazine	(E)-2-diethoxyphosphoryl-3-[5-(2,4-difluorophenyl)furan-2-yl]prop-2-enenitrile
4',4',6'-trimethyl-N-phenylspiro [1,3,4-oxadiazole-5,2'-7-oxabicyclo[4.1.0]heptane]-2-imine	1-(3,8,8-trimethyl-6,7-dihydro-5H-naphthalen-2-yl)ethanone
1-[(4-chlorophenoxy)-(2-ethylaziridin-1-yl)phosphoryl]-2-ethylaziridine	4-(2-chloroacetyl)-6,7-dimethyl-1,3-dihydroquinoxalin-2-one
6-(2-chloroanilino)-4-oxo-2-pyridin-4-yl-2,3-dihydro-1,3-thiazine-5-carbonitrile	5-methylsulfanyl-6-phenylsulfanyl-2H-1,2,4-triazin-3-one
N-[(1Z)-{[3-(2,6-dichlorophenyl)-5-methyl-1,2-oxazol-4-yl]formohydrazido}(methylsulfanyl) methylidene]benzenesulfonamide	3-dibutoxyphosphoryl-3,5,5-trimethylcyclohexan-1-one
(Z)-2-[4-(3-ethyl-5,5,8,8-tetramethyl-6,7-dihydronaphthalen-2-yl)-1,3-thiazol-2-yl]-N'-hydroxyethenimidamide	[cyclohexen-1-yloxy(phenyl)phosphoryl]benzene
2-[2-[(6-oxobenzo[c]isochromen-2-yl)carbamoyl]phenyl]benzoic acid	5-morpholin-4-yl-1,5-di(phenyl)pent-1-en-3-one
6-penta-1,3-diynylpyran-2-one	2-arsonobenzoic acid
Glutathione	5,5-dimethyl-6H-benzo[c]acridin-6-ol
2-carbazol-9-ylbenzoic acid	Benzotriazole,4,5,6,7-tetrachloro- (6CI,7CI); 4,5,6,7-Tetrachlorobenzotriazole
5-(2-naphthylmethylene)-1,3-dihydropyrimidine-2,4,6-trione	ethyl 4-[(2-hydroxynaphthalen-1-yl)methyl]piperazine-1-carboxylate
Barbituric acid	1-(3-methylphenoxy)-3-[4-(4-methylphenyl)piperazin-1-yl]propan-2-ol
2-Methoxy-5H-pyrido(3',2':5,6)(1,4)thiazino(2,3-b)quinoxaline	3-[(3-ethoxy-4-hydroxyphenyl)methylidene]-5-(4-methoxyphenyl)furan-2-one
(1-tert-butyl-5-phenylpyrrol-3-yl)-phenylmethanone	diethyl 4-(4-methoxyphenyl)-2,6-dimethylpyridine-3,5-dicarboxylate
ethyl (E)-2-cyano-3-[2-(ethylamino)pyrazolo[1,5-a]pyridin-3-yl]prop-2-enoate	[2-(4-chlorophenyl)quinolin-4-yl]-piperidin-2-ylmethanol
N-(4,7-Dihydroxy-8-methyl-2-oxo-2H-chromen-3-yl)-2,2-dimethylchromane-6-carboxamide	6-bromo-N-naphthalen-1-yl-1,3-benzodioxole-5-carboxamide
N-(2,5-dimethoxyphenyl)-2-hydroxydibenzofuran-3-carboxamide	N,N-dimethyl-1,3-dioxoisoindole-2-sulfonamide
diethyl 2-acetamido-2-dodecylpropanedioate	
4-oxo-4-pyren-1-ylbutanoic acid	

1,1,3,3-tetra(phenyl)urea	
(4E)-4-(3H-1,3-benzothiazol-2-ylidene)-3-hydroxycyclohexa-2,5-dien-1-one	

NCI Natural Products

**Hits from DSF not tested in Biolayer Interferometry**	**Hits from DSF tested in Biolayer Interferometry**
(1R,4aS,10aR)-1,4a-dimethyl-7-(propan-2-yl)-1,2,3,4,4a,9,10,10a-octahydrophenanthrene-1-carboxylic acid	(5E,9E)-4,12-dihydroxy-2,6,10-trimethyl-14-methylidene-16-oxabicyclo[11.3.1]heptadeca-5,9-dien-15-one
3aR,5R,5aR,8aR,9S,9aS)-9-hydroxy-5,8a-dimethyl-1-methylidene-3a,4,5,5a,9,9a-hexahydroazuleno[6,7-b]furan-2,8-dione	Methyl (1aS,1bS,3aR,4R,7aS,9aS)-1b-hydroxy-4,7a-dimethyl-9-oxo-9a-propan-2-yl-2,3,3a,5,6,7-hexahydro-1aH-phenanthro[1,2-b]oxirene-4-carboxylate
13-Methoxy-3,3-dimethyl-11-(3-methyl-2-butenyl)-7a,12a-dihydro-3H,7H-(1)benzofuro(3,2-c)pyrano(3,2-g)chromen-10-ol	sodium 2-[(3S,6R)-6-[[(5R,7R,9R,10R)-2-[5-[(3S,5R)-5-[(2S,3S,5R,6R)-6-hydroxy-6-(hydroxymethyl)-3,5-dimethyloxan-2-yl]-3-methyloxolan-2-yl]-5-methyloxolan-2-yl]-9-methoxy-2,4,10-trimethyl-1,6-dioxaspiro[4.5]decan-7-yl]methyl]-3-methyloxan-2-yl]propanoate

Maybridge Hitfinder Library

Hits from DSF tested in Biolayer Interferometry
6-nitro-2-(2-thienyl)-1,2,3,4-tetrahydroquinazolin-4-one
N1-(5,7-dimethyl[1,8]naphthyridin-2-yl)-4-(trifluoromethyl)benzamide
3-(2,3-dihydro-1,4-benzodioxin-6-ylamino)-2(propylsulfonyl)acrylonitrile
2-{[4-(5-nitropyridin-2-yl)piperazino]carbonyl}cyclobutanecarboxylic acid
N1-(2-{2-[2-(acetylamino)phenoxy]ethoxy}phenyl)acetamide
2-benzo[b]furan-2-yl-3-(4-fluorophenyl)-1,3-thiazolan-4-one
N1-(2,4-dichlorophenyl)-2-hydrazino-2-oxoacetamide
ethyl 2-methyl-4-(morpholinosulfonyl)-1,5-diphenyl-1H-pyrrole-3-carboxylate
N-{3-[3-(acetylamino)phenoxy]propyl}-3-(2-chloro-6-fluorophenyl)-5-methyl-4-isoxazolecarboxamide
5-methyl-2-nitro-N'-[(3,4,5-trimethoxyphenyl)methylene]benzohydrazide
N-[2-hydroxy-3-(4-methoxyphenoxy)propyl]cyclobutanecarboxamide
N4-(3,4-dimethoxyphenethyl)-6-methylpyrimidine-2,4-diamine
3,5-di(acetyloxy)-2-(1,3-benzothiazol-2-yl)tetrahydro-2H-pyran-4-yl acetate
N1-[2,2,2-trifluoro-1-(4-fluoroanilino)-1-(trifluoromethyl)ethyl]-2-ethoxybenzamide
N3-(3,4-dichlorobenzyl)-2-{[3-(trifluoromethyl)phenyl]thio}nicotinamide
2-({[3-(4-chlorophenyl)-1,2,4-oxadiazol-5-yl]methyl}sulfanyl)acetic acid
5-chloro-2-[(2-chloro-6-fluorobenzyl)sulfanyl]-1-isopropyl-1H-benzimidazole
1-bromo-2-methoxynaphthalene
3-[(4-chlorophenyl)sulfonyl]-1,3-thiazolane-2-carbohydrazide
2-[(5-{[(1-naphthylmethyl)thio]methyl}-4-phenyl-4H-1,2,4-triazol-3-yl)thio]-3-nitropyridine
[1-(4-methoxyphenyl)-5-methyl-1H-pyrazol-4-yl](piperidino)methanone
methyl 3,5-dibromo-2-(2-methoxy-2-oxoethoxy)benzoate
1-[3-({[1-(3,4-dimethoxyphenyl)-1H-1,2,3,4-tetraazol-5-yl]thio}methyl)-4-methoxyphenyl]ethan-1-one
Trisodium 8-hydroxypyrene-1,3,6-trisulfonate
4-cyclohexyl-1-methyl-2,3,4,5-tetrahydro-1H-1,4-benzodiazepine-2,5-dione
2-[(2-methyl-3-furyl)carbonyl]-3-[(methyl{4-[(5-nitropyridin-2-yl)oxy]phenyl}oxo-lambda~6~-sulfanylidene)amino]acrylonitrile
2-chloro-6-fluoro-N'-[2-(trifluoromethyl)phenyl]benzohydrazide
4-amino-2-{[3-(trifluoromethyl)benzyl]sulfanyl}-5-pyrimidinecarboxylic acid
methyl 3-{1-[3-hydroxy-4-(hydroxymethyl)tetrahydrofuran-2-yl]-2,4-dioxo-1,2,3,4-tetrahydropyrimidin-5-yl}acrylate
5-nitro-2-{[5-(2-thienyl)-4H-1,2,4-triazol-3-yl]thio}-1,3-thiazole
2-(2,3-dihydro-1,3-benzoxazol-2-yl)-2,3-dihydro-1,3-benzoxazole
2-[(2-methylimidazo[1,2-a]pyridin-3-yl)carbonyl]hydrazine-1-carboxamide
N1-[2-(difluoromethoxy)benzylidene]-4-chloroaniline
4-(octyloxy)benzoic acid
2-(2,3-dihydro-1,3-benzoxazol-2-yl)-2,3-dihydro-1,3-benzoxazole
N2-(2,6-dimethoxy-3-pyridyl)-3-bromo-5-chloro-2-thiophenesulfonamide
N'-{[3-(4-chlorophenyl)-1-phenyl-1H-pyrazol-4-yl]methylene}-2-{[4-phenyl-5-(4-pyridinyl)-4H-1,2,4-triazol-3-yl]sulfanyl}acetohydrazide
phenyl 3-aminopyrazine-2-carboxylate
2-thienyl[3-(trifluoromethyl)-1H-pyrazol-1-yl]methanone

methyl 2-{3-[3-(trifluoromethyl)phenyl]triaz-1-enyl}benzoate
3-{2-[(4-chlorophenyl)thio]-5-nitrophenyl}-1H-pyrazole
N1-(tert-butyl)-9-oxo-9H-1-fluorenecarboxamide
1-phenyl-1H-pyrazole-5-carboxylic acid
2-[(4-chlorobenzyl)oxy]-6-fluorobenzonitrile
ethyl 5-[(2,1,3-benzoxadiazol-4-ylsulfonyl)amino]-2-piperidinobenzoate
3-{[({[2-(trifluoromethoxy)phenyl]methylene}amino)oxy]carbonyl}thiophene
O1-{3-[2-oxo-3-(trifluoromethyl)-1,2-dihydropyridin-1-yl]propanoyl}-4-(trifluoromethoxy)benzene-1-carbohydroximamide
N1-(2,4-difluorobenzyl)-4-fluorobenzamide
6,9-dimethoxy-11H-indeno[1,2-b]quinoxalin-11-one
tert-butyl N-[(1-benzyl-4-piperidinyl)methyl]carbamate
**(4-benzhydrylpiperazino)(1,5-dimethyl-1H-pyrazol-3-yl)methanone (ccrp2)**
N-[(4-chlorobenzoyl)oxy](4-chlorophenyl)dioxo-lambda~6~-sulfanecarboximidoyl cyanide
5-chloro-3-{[(2,4-dinitrophenyl)thio]methyl}benzo[b]thiophene
3-[4-(tert-butyl)-3-({5-[3-(trifluoromethyl)phenyl]-2H-1,2,3,4-tetraazol-2-yl}methyl)phenyl]-1,2,4-oxadiazole
N2-(2,6-dimethoxy-3-pyridyl)-3-bromo-5-chloro-2-thiophenesulfonamide
5-(2,4-dichlorobenzyl)-2-mercapto-4,6-dimethylnicotinonitrile
2-(4-fluorophenyl)-5-(4-methyl-1,2,3-thiadiazol-5-yl)-1,3,4-oxadiazole
5-(4-methylphenyl)-1,3-oxazole
ethyl 4-{[(2,5-dichloro-3-thienyl)carbonyl]amino}tetrahydro-1(2H)-pyridinecarboxylate
3-chloro-N-(2-quinolinyl)-1-benzothiophene-2-carboxamide
N1-[4-(tert-butyl)phenyl]-2-{[5-(4-chloro-1-methyl-1H-pyrazol-3-yl)-4,5-dihydro-1,3,4-thiadiazol-2-yl]thio}acetamide
methyl 1-methyl-2-{[3-(trifluoromethyl)anilino]carbonyl}-1-hydrazinecarbodithioate
N-(3-cyano-4,5-dihydronaphtho[1,2-b]thiophen-2-yl)acetamide
5-[4-(tert-butyl)phenyl]-1-phenyl-1H-1,2,4-triazol-3-yl acetate
N1-(3-fluorophenyl)-2-(tert-butyl)hydrazine-1-carbothioamide
5-isopropyl-3,8-dimethylazulene-1-carbaldehyde 1-(4-nitrophenyl)hydrazone
2-[2-chloro-5-(trifluoromethyl)anilino]-5-methoxybenzoic acid
1-(2-phenyl-1,3-thiazol-4-yl)ethan-1-one 1-[4-(4-chlorophenyl)-1,3-thiazol-2-yl]hydrazone hydrobromide
N-{2-[(2,4-dichlorobenzyl)thio]ethyl}-4-methyl-1,2,3-thiadiazole-5-carboxamide
N1-(3-morpholinopropyl)-2-aminobenzamide
O1-[(3,5-dimethylisoxazol-4-yl)carbonyl]-2-[3-(trifluoromethyl)-1H-pyrazol-1-yl]ethanehydroximamide
4-[({5-[4-(tert-butyl)phenyl]-4H-1,2,4-triazol-3-yl}thio)methyl]-2-methyl-1,3-thiazole
